# CDKN2AIP is critical for spermiogenesis and germ cell development

**DOI:** 10.1186/s13578-022-00861-z

**Published:** 2022-08-21

**Authors:** Yuming Cao, Qi Sun, Zhenlie Chen, Jing Lu, Ting Geng, Ling Ma, Yuanzhen Zhang

**Affiliations:** 1 Reproductive Medicine Center, Zhongnan Hospital of Wuhan University, Wuhan, China; 2 Hubei Clinical Research Center for Prenatal Diagnosis and Birth Health, Wuhan, 430071 Hubei People’s Republic of China; 3 Department of Obstetrics and Gynecology, Zhongnan Hospital of Wuhan University, Wuhan, 430071 Hubei People’s Republic of China; 4 Hubei Provincial Key Laboratory of Developmentally Originated Disease, School of Basic Medical Sciences, Wuhan University, Wuhan, 430070 Hubei People’s Republic of China

**Keywords:** Spermiogenesis, Germ cell development, DNA double-strand break repair, Protamine replacement, Male infertility

## Abstract

**Background:**

As a member of RNA-binding protein, CDKN2AIP has been shown to play a critical role in stem cell pluripotency and somatic differentiation. Recent studies indicate that *Cdkn2aip* is essential for spermatogonial self-renewal and proliferation through the activating Wnt-signaling pathway. However, the mechanisms of how *Cdkn2aip* regulate spermatogenesis is poorly characterized.

**Results:**

We discovered that the CDKN2AIP was expressed in spermatocyte as well as spermatids and participated in spermiogenesis. *Cdkn2aip*^*−/−*^ mice exhibited multiple sperm head defects accompanied by age dependent germ cell loss that might be result of protamine replacement failure and impaired SUN1 expression. Loss of *Cdkn2aip* expression in male mice resulted in synapsis failure in 19% of all spermatocytes and increased apoptosis due to damaged DNA double-strand break (DSB) repair and crossover formation. In vitro, knockdown of *Cdkn2aip* was associated with extended S phase, increased DNA damage and apoptosis.

**Conclusions:**

Our findings not only identified the importance of CDKN2AIP in spermiogenesis and germ cell development, but also provided insight upon the driving mechanism.

**Supplementary Information:**

The online version contains supplementary material available at 10.1186/s13578-022-00861-z.

## Introduction

During spermatogenesis, spermatogonia undergo mitosis and differentiate into spermatocytes. Primary spermatocytes then receive necessary modification and eventually become secondary spermatocytes through preleptotene, leptotene, zygotene, pachytene, and diplotene meiotic steps [1, 2]. Subsequently, secondary spermatocytes need to undergo dramatic morphological changes before differentiating into mature spermatozoa [3]. This complicated process is characterized by gene expression and cellular morphology alterations. Meiotic or spermatogenesis disorders often lead to terrible consequences such as infertility [4]. During meiosis, telomeres attach to the nuclear envelope (NE) to promote homologous chromosome moving, pairing, synapsis, and recombination. In mammals, the meiosis-specific linker of nucleoskeleton and cytoskeleton (LINC) complex, which is consisted of KASH5 and SUN1, provides the binding site for telomeres [5, 6]. Previous studies showed that knockout of either of these two genes in mice resulted in homologous pairing defects and meiotic arrest, suggestive of an essential role of the meiotic LINC complex in prophase progression [7, 8]. Sad1/UNC84 homology proteins are a family of nuclear membrane proteins that share a conserved C terminus the SUN domain [9]. SUN proteins as a component of the LINC complex that responsible for various important cellular functions, such as mechano-transduction, cellular signaling, nuclear anchorage, and positioning [10]. The meiosis-specific protein SUN1 provides the binding site for telomeres for transmission of cytoskeletal forces to regulate telomere dynamics [11]. Many homologous chromosomes in *Sun1*^*−/−*^ mice fail to pair and synapse during pachynema, partially completed synaptonemal complexes are still present in pachytene-like staged meiocytes [12]. Although an overall molecular model has been built, the detailed mechanisms underlying the nature of SUN1’s regulation and how telomeres are protected during meiosis still remains unknown.

Spermiogenesis is a critical, post-meiotic phase of gametogenesis defined as the differentiation of spermatids into spermatozoa [13]. During this process, impressive degree of morphological changes and gene expression alterations occur. Chromatin remodeling is an important epigenetic process during germ cell development and essential for proprt sperm maturation [14]. This process is regulated by the performance of specialized factors, in which most core histones are initially hyperacetylated and then replaced by transition proteins (TNP1 and TNP2), which are themselves later replaced by protamines (PRM1 and PRM2) [15]. In sperm nuclei, the DNA-protamine complex, which represents a higher order of DNA packaging, compacts, stabilizes, and protects the haploid genome. Therefore, aberrations in protamine expression and formation are often associated with infertilities. However, the mechanisms behind histone displacement are unclear.

The previous study identified CDKN2AIP as a novel ARF-binding protein in p53 pathway [16]. Later studies showed that CDKN2AIP could interact with p53 directly and facilitate p53 activation independent of ARF [17]. In addition, knocking down CDKN2AIP expression results in aneuploidy, DNA damage, and mitotic catastrophe, resulting in apoptosis via the ATR/CHK1 pathway [18]. In the meantime, overexpression of CDKN2AIP impaired cell proliferation and resulted in senescence by activation of p53-HDM2-p21 pathway [19]. Interestingly, our recent work found that CDKN2AIP is expressed in almost all human tissues but highest in testis, where its expression was associated with testicular tumor suppression. However, it remains elusive whether *Cdkn2aip* has a specific role in spermatogenesis.

To decipher the biological importance of *Cdkn2aip* and its molecular actions, we generated *Cdkn2aip* knock-out mice and examined the role of *Cdkn2aip* in spermiogenesis.

## Materials and methods

### Cytoplasmic separation and Western blot analysis

Mouse testicular tissue was lysed using nuclear and cytoplasmic protein extraction kit (Beyotime, P0027) according to the manufacturer’s instructions. The lysates were ultracentrifuged at 12000 *g* for 10 min at 4 °C. The supernatants and remaining sediment were collected separately. The concentration of protein in the sediment and supernatants were measured using a bicin-choninic acid assay (Beyotime Biotechnology, P0012S). For western blot assay, 50 μg of protein samples was loaded on 10% SDS-PAGE gel and runed 1.5 h at 100 V before transferring to PVDF membranes. The antibodies used were as follows. Rabbit anti-CDKN2AIP (1:1000, Proteintech, Cat No.16615-1-AP), Mouse anti-SYCP3 (1:1000, Abcam, Cat No. ab97672), Rabbit anti-histone H3(1:1000, Abcam, Cat No.ab1791), Rabbit anti-PRM1(1:1000, Affinity, Cat No.DF5045), Rabbit anti PRM2(1:1000, Proteintech, Cat No.14500-1-AP), Rabbit anti-SUN1(1:1000, Abcam, Cat No.ab103021), Mouse anti-GAPDH (1:10,000, ABclonal, Cat No.AC002), Goat Anti-Rabbit IgG H&L (HRP) (1:8000, Abcam, ab6721), Rabbit Anti-Mouse IgG H&L (HRP) (1:8000, Abcam, ab6728).

### Immunohistochemistry

Paraffin-embedded fixed mouse tissues were deparaffinized and rehydrated using xylene and ethanol. Antigen retrieval was achieved by placing the slides in boiling citrate buffer, pH 6.4 for 20 min. After cooling at room temperature for 20 min, slides were rinsed with ddH_2_O and TBST (Tris-buffered saline with 0.1% Tween-20) successively. Endogenous peroxides were quenched by 3% H_2_O_2_ treatment for 10 min. The slides were blocked with background eraser solution (Biocare Medical, Concord, CA, USA) containing 10% goat serum for 5 min. Primary antibody diluted with TBST was applied and slides were incubated in a humidified chamber for 1 h. After rinsing with TBST, a biotinylated anti-rabbit IgG secondary antibody was added followed by incubation with a peroxidase-based Vectastain avidin–biotin complex (ABC, Vector Laboratories, Burlingame, CA, USA). Color was developed using DAB (3,3′-diaminobenzidine) substrate-chromogen. The nucleus was counterstained with Methyl Green. The antibodies used were as follows. Rabbit anti-CDKN2AIP (1:100, Proteintech, Cat No.16615-1-AP), Mouse anti-SYCP3 (1:100, Abcam, ab15093), Mouse anti-γH2AX (1:200, Sigma, Cat No. 05-636), Rabbit anti-53BP1(1:100, Homemade), Rabbit anti-RAD51(1:100, Homemade), Rabbit anti-MLH1(1:100, Homemade), Rabbit anti-HORMAD1(1:100, Homemade), Rabbit anti-histone H3(1:1000, Abcam, Cat No. ab1791), Rabbit anti-TNP1(1:100, Proteintech, Cat No. 17178-1-AP), Rabbit anti-PRM1(1:100, Affinity, Cat No.DF5045), Rabbit anti PRM2(1:100, Proteintech, Cat No.14500-1-AP), Rabbit anti-SUN1(1:100, Abcam, Cat No. ab103021),Goat Anti-Rabbit IgG H&L (Alexa Fluor^®^ 488) (1:400, Abcam, ab150077), Goat Anti-Rabbit IgG H&L (Alexa Fluor^®^ 594) (1:400, Abcam, ab150080).

### Total RNA extraction and RT–PCR analysis

Total RNA was collected from *Cdkn2aip*^+*/*+^ and *Cdkn2aip*^*−/−*^ mice testicular tissue using the TRIzol reagent (Sangon, Shanghai, P. R. China) and immediately reverse-transcribed using revertAid RT reverse transcriptase kit (Thermo Scientific™, K1691) according to the manufacturer’s protocol. The expression of *Cdkn2aip*, and *Sun1* were amplified by PCR using β-actin as housekeeping gene. The primers used in this study are listed in Additional file 6: Table S1.

### Mouse model

By co-injecting sgRNA and Cas9 mRNA into fertilized eggs of C57BL/6 mice, mouse model with a 3687-base deletion was generated. The founders were genotyped by PCR followed by DNA sequencing analysis. Intercrossing of *Cdkn2aip* heterozygous mice yielded healthy offspring at Mendelian ratios. The use of mice was approved by the Animal Ethics Committee of the School of Medicine, Wuhan University. All animal care protocols and experiments were reviewed and approved by the Animal Use Committee of the School of Medicine, Wuhan University.

### Histological analysis

The testes and epididymides were collected from mouse model and fixed in Bouin’s fixative solution for 24 h before embedding in paraffin. Then, slices of 5 μm thickness were cut from the block and stained with Hematoxylin and Eosin.

### Epididymal sperm count and morphological analysis

The cauda epididymis was dissected from adult mice. Sperm was squeezed out from the cauda epididymis and incubated for 30 min at 37 °C under 5% CO_2_. The medium was then diluted at 1:500 and transferred to a hemocytometer for counting. Fixed sperm were spread on precoated slides for morphological observation by Barclay stain (Ultra-Fast Modified Papanicolaou Stain Kit, D022-1-3).

### Transmission EM

Sperms were fixed with 2.5% glutaraldehyde in 0.2 M cacodylate buffer overnight. After rinsing in 0.2 M PB, the tissue was cut into small pieces, approximately 1 mm^3^, and immersed in 1% OsO4 in 0.2 M cacodylate buffer for 2 h at 4 °C. Then, the samples were dehydrated through a graded ethanol series and embedded in resin. Ultrathin sections were obtained and stained with uranyl acetate and lead citrate and then observed using a JEM-1400 transmission electron microscope.

### In vitro fertilization assays

Superovulation from C57BL/6 mice were achieved by intraperitoneal injections of 5 international units (IU) of pregnant mare serum gonadotropin and 5 IU of human chorionic gonadotropin (hCG) 48 h later. The follicles were recovered 24 h after hCG injection. Spermatozoa were isolated from the cauda epididymis of each male, capacitated in TYH medium at 37 °C for 1 h before adding to the follicles. The cells were incubated for 6 h at 37 °C in 5% CO_2_ covered with mineral oil. After 24 h, two‐cell stage embryos were counted.

### Immunofluorescence

Tissue sections were processed for antigen retrieval with citrate buffer and permeabilized in cold acetone for 5 min. After blocking with 3% BSA + 10% goat serum in PBS for 1 h at room temperature (RT), sections were incubated with primary antibody overnight at 4 °C and then treated with Alexa Fluor 488-, Alexa Fluor 594 secondary antibodies for 1 h at RT. Nuclei were counterstained with DAPI (Vector Laboratories). Images were captured with a LSM 880 confocal microscope (Zeiss).

### TUNEL assay

Apoptotic cells were analyzed by Fluorometric TUNEL System (In Situ Cell Death Detection Kit, POD) as previously described [20]. Paraffin-embedded testis sections were incubated with TUNEL reaction buffer under humidified atmosphere for 60 min at 37 °C, then rinsed with PBS for three times. The nuclei were stained with DAPI. TUNEL-positive cells were identified by the emission of green fluorescence.

### RNA-seq

Total RNA was extracted from testes samples of 6 weeks old *Cdkn2aip*^+*/*+^ and *Cdkn2aip*^−/−^ mice as described previously. Prior to sequencing, the total RNA was subject to DNase I treatment (DNase-free, Ambion) to remove trace genomic DNA and the RNA quality was assessed with an Agilent Bioanalyzer 2000 platform. The library preparation and sequencing were completed by the Novogene. Starting with 5ug (≥ 300 ng/uL) of RNA the ribosomal RNA was depleted using Life Technologie’s Ribo Minus Eukaryote System v2 per manufacturer’s instructions. The library preparation was then completed using Life Technologie’s Ion Total RNA-Seq Kit v2 Library Kit and Ion Xpress RNA-Seq Barcodes, following manufacturer’s instructions. Library size and quantitation was established using the Agilent High Sensitivity DNA Kit. Templated ISPs were prepared using Life Technologie’s Ion PI Template OT2 200 Kit version 2, following manufacturer’s instructions. The sequencing was conducted on a Life Technologie’s Ion Torrent Proton Sequencer using the Life Technologie’s Ion PI Sequencing 200 Kit version 2 and Life Technologie’s Ion PI v2 Chip, per manufacturer’s instructions. The RNA library sequencing and data analysis process was described in Additional file 4: Fig. S4A.

### Co-immunoprecipitation followed by mass spectrometry (IP-MS)

Testis of 8 weeks old male *Cdkn2aip*^+*/*+^ and *Cdkn2aip*^*−/−*^ mice were collected and lysed in IP buffer (Beyotime P0013). The cellular membrane debris was finally removed by centrifugation at 4 °C for 30 min at 12000 rpm. CDKN2AIP antibody was incubated with testicular lysate at 4 °C for 6 h. 50 μl protein A beads were added into the reaction and incubated at 4 °C overnight with gentle shaking. The bead-complex was washed for three times with incubated buffer for 5 min/each at 4 °C. The protein complex was finally eluted off the beads into 2 × SDS loading buffer and loaded to 10% gradient SDS PAGE gel to visualize all protein bands through silver staining followed by mass spectrometry, as described in Additional file 4: Fig S4B.

### Biolayer interferometry (BLI)

A His-fusion protein of CDKN2AIP were diluted in assay buffer containing 50 mM Tris buffer solution (PH 7.2), 25 mM NaCl, 0.02% polysorbate-20 and 0.1% Bovine Serum Albumin.CDKN2AIP protein was first bound to the ssDNA and rinsed by assay buffer. The assay was performed in solid black 96-well plates (Greiner Bio-One, cat.no.655209), using agitation set at 1000 rpm, and temperature set at 30 °C.

### RIP-Seq

The TM4 cells were rinsed with PBS twice before adding trypsin for digestion. The cells were collected and rinsed with pre-cooled 2 ml–0.01 mol PBS for three times. The cells were then resuspended with a complete Lysis Buffer equal to the cells’ volume and incubated on ice for 5 min. 50 μl protein A/G Agrose magnetic beads were added to each sample, then 500 μl RIP Wash Buffer was added and gently voracized, after briefly centrifuged (3000–5000 *g* for 1 min), the supernatant was removed. 100 μl RIP Wash Buffer was added and 5 μg antibody was added to each group and incubate at room temperature for 30 min on a rocker. 500 μl RIP Wash Buffer was added and centrifuged briefly to remove the supernatant. 900 μl RIP Immunoprecipitation Buffer was added to each sample. Cell lysates were rapidly thawed and centrifuged at 4, 14,000 RPM for 10 min. 100 μl of supernatant was collected from each IP sample, resulting in a final volume of 1 ml. 10 μl cell lysate supernatant was taken into a new Nucleus Free EP tube as 10% Input. The remaining IP sample was incubated overnight, and supernatant was removed after centrifugation. 500 μl precooled RIP Wash Buffer was added to wash the sample repeated 5 times. The integrity and purity of RNA eluted were assessed using an Agilent Bioanalyzer.

### Cell cycle analysis

Cells were harvested and fixed in freshly prepared pre-cooled 70% ethanol overnight at 4 °C. Cells were centrifuged at 1000 *g* for 5 min and stained with propidium iodide (PI, Beyotime) at 37 °C for 30 min. Cell cycle phases were determined by FACS analysis and the data were processed using ModFit LT.

### Ionizing radiation

To induce DNA damage, TM4 cells were cultured under feeder-free condition and treated with a dose of 5 Gy irradiation by Gamma cell 40 Exactor. TM4 without treatment, or 2 h, 12 h and 24 h later after treatment were collected for analysis.

### Statistical analysis

All data were reported as mean ± SD in the figure legends. Two-tailed unpaired Student’s *t* was used to determine the significance (*P < 0.05; **P < 0.01; ***P < 0.001). All diagrams were drawed with Prism 8.0 (GraphPad Software, La Jolla, CA, USA).

## Result

### CDKN2AIP preferentially expressed in the testis and mainly localized to the nuclei of spermatocyte and spermatids

**Fig. 1:**
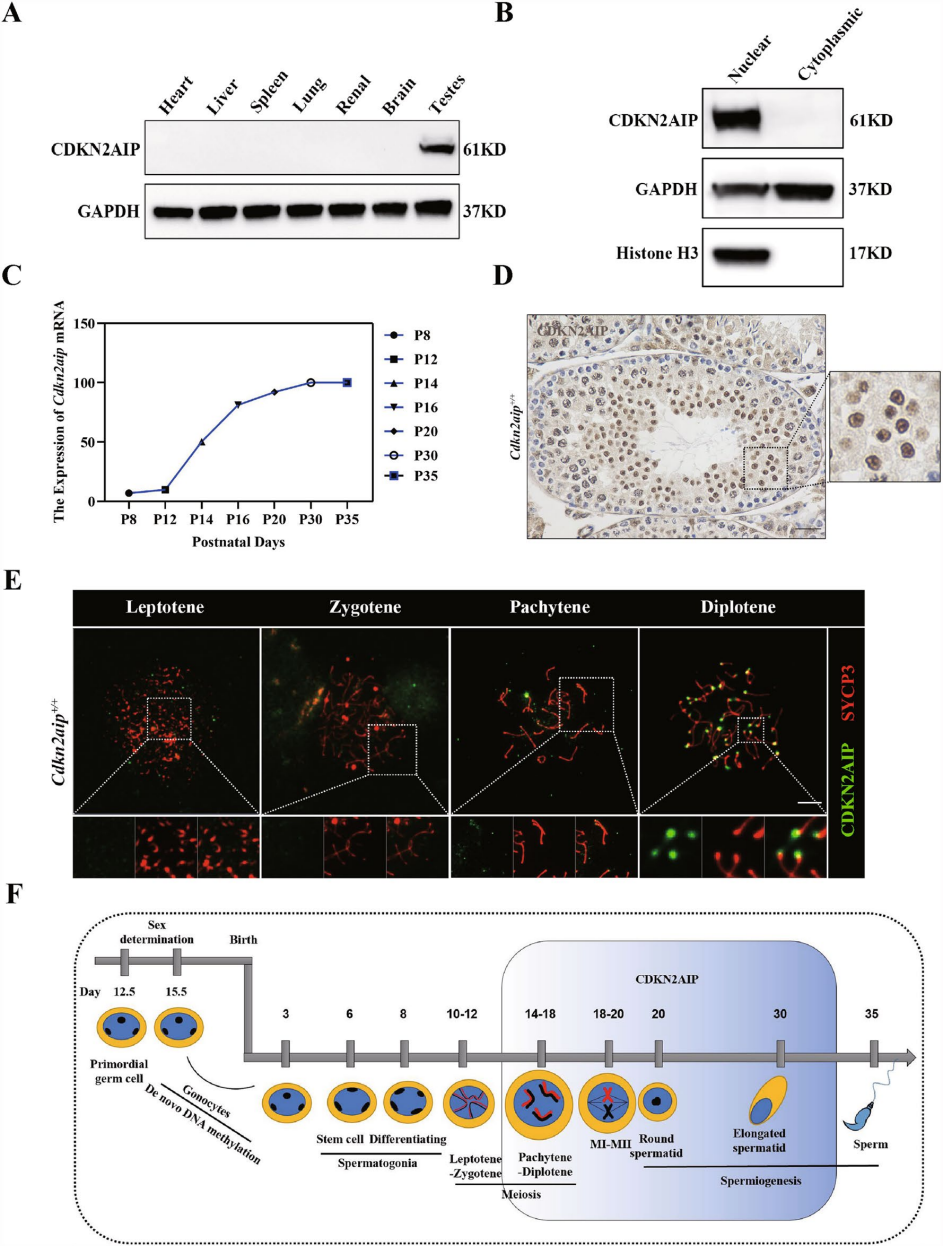
Expression profiles of CDKN2AIP during testicular development and spermatogenesis in mice. **A** Western blot analysis was performed using 20 μg protein for CDKN2AIP in various mouse tissues. GAPDH is serves as a loading control. **B** Isolation of nuclear protein and cytoplasmic protein from 8-week age testicular tissue by western blotting assay. Histone H3 is a nuclear-associated protein, and GAPDH is ubiquitous in the nucleus and cytoplasm. **C** Expression of *Cdkn2aip* mRNA during postnatal testicular development. Levels of *Cdkn2aip* mRNAs in developing testes at postnatal day 8 (P8), P10, P14, P16, P30 and P35 were analyzed using qPCR, β-actin is serves as a housekeeping gene. **D** Localization of CDKN2AIP in the *Cdkn2aip*^+*/*+^ adult testis, as detected by immunohistochemistry. CDKN2AIP is stained brown. Scale bar, 50 μm. **E** Immunostaining of CDKN2AIP (green) and SYCP3 (red) on chromosome spreads of spermatocytes from P35 *Cdkn2aip*^+*/*+^ testes, n = 3 mice for each group. Scale bars, 10 μm. **F** Schematic diagram showing the proposed model of CDKN2AIP expression pattern during spermatogenesis in mice

Our data indicated that the *Cdkn2aip* mRNA and protein was expressed with the highest level in testis and was almost undetectable in ovaries or other tissues (Fig. 1A and Additional file 1: Fig. S1A). As shown in Fig. 1B and D, CDKN2AIP is a nuclear binding protein and most expression of it located near the nuclei of spermatocyte and spermatids. During postnatal testicular development, *Cdkn2aip* mRNA expressions started to increase drastically at postnatal day 14, simultaneously with occurrence of pachytene spermatocytes, and peaked at P30 (Fig. 1C). Next, we performed chromosome spread immunofluorescence staining of mouse testes, and discovered that CDKN2AIP was specificity concentrated at the chromosome single ends in pachytene and diplotene spermatocyte (Fig. 1E). During the second stage of meiosis when haploid chromatids were produced, CDKN2AIP was highly expressed at the nuclei and periphery area of round sperm (Fig. 1D). These results shows that CDKN2AIP is predominantly localized to the nuclei of spermatocytes and spermatids from about 14 to 30 days postpartum, which may play an important role in spermatogenesis (Fig. 1F).

### Targeted knock-out of the *Cdkn2aip* Gene resulted in age-dependent infertility in male mice

**Fig. 2:**
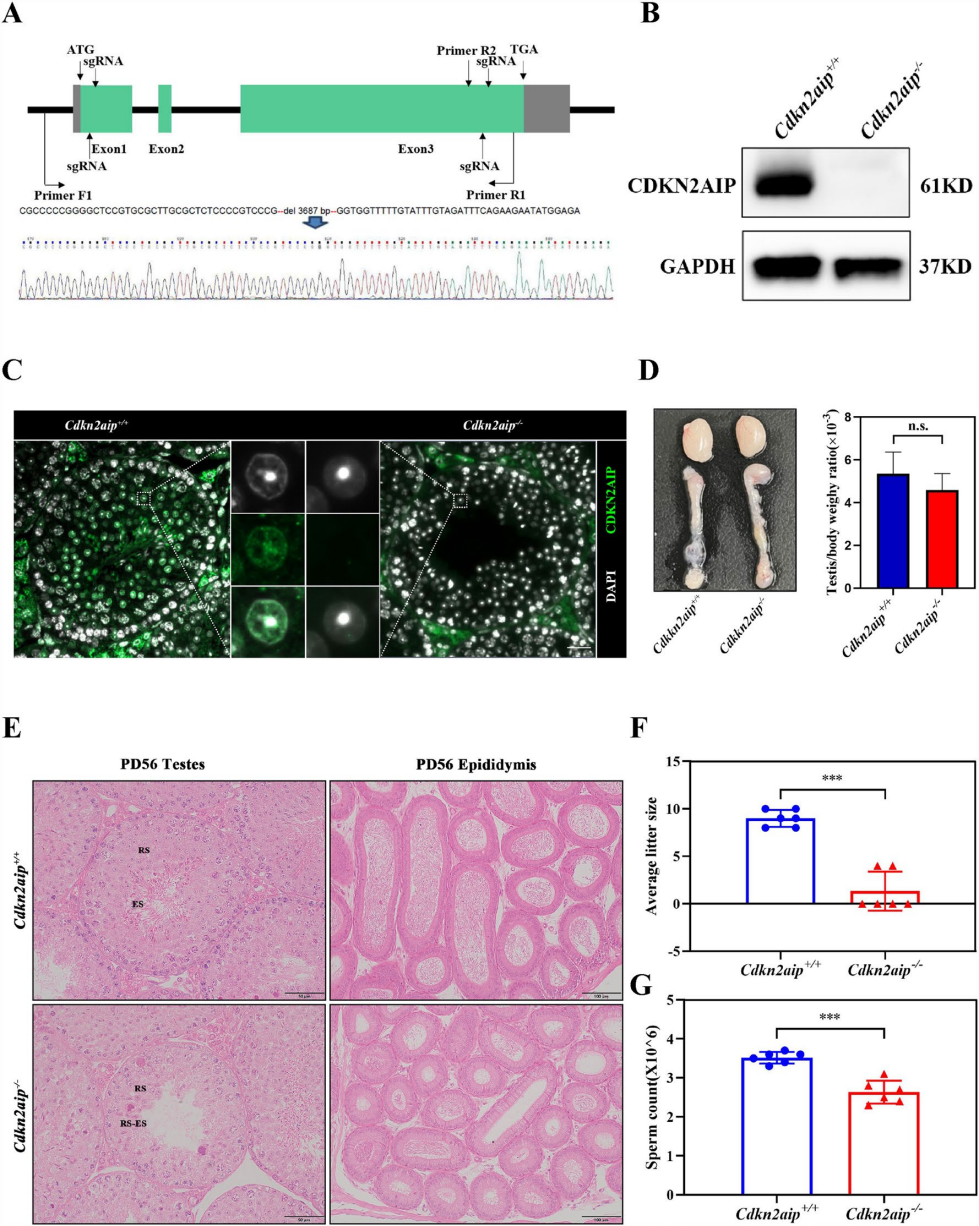
Deletion *Cdkn2aip* results in abnormal spermatogenesis and increased male infertility. **A** Schematic representation of the genome editing strategy at the *Cdkn2aip* locus showing the sgRNAs (arrows), the corresponding coding exons (green thick lines). Sequencing results showed that 3687 bp fragment deleted. **B** Western blotting using the rabbit anti-CDKN2AIP antibody confirms the lack of full-length CDKN2AIP proteins in testis lysates from 8-week-old *Cdkn2aip*^*−/−*^ mice. GAPDH is serves as loading control. **C** Immunofluorescent staining of a frozen testis section from 8-week-old *Cdkn2aip*^+*/*+^ and *Cdkn2aip*^*−/−*^ by CDKN2AIP (green) antibodies. Nuclei are labeled by DAPI (blue). Scale bar, 50 μm. **D** Representative images of testes from 8-week-old *Cdkn2aip*^*−/−*^ and *Cdkn2aip*^+*/*+^ mice, and average testes/body weight ratio of 8-week-old *Cdkn2aip*^+*/*+^ and *Cdkn2aip*^*−/−*^, Data are presented as mean ± S.D. Student’s *t* test, n.s. *P* > 0.05. **E** Testicular and epididymal sections of 8-week-old *Cdkn2aip*^+*/*+^ and *Cdkn2aip*^*−/−*^ were stained with H&E. Scale bar, 50 μm. **F** Litter sizes from wild type females mated with either *Cdkn2aip*^+*/*+^ and *Cdkn2aip*^*−/−*^. Data are presented as mean ± S.D. Student’s *t* test; ***P < 0.001. **G** Relative number of epididymal sperm number of 8-week-old *Cdkn2aip*^+*/*+^ and *Cdkn2aip*^*−/−*^ mice (n = 6). Data are presented as mean ± S.D. Student’s *t* test; ***P < 0.001

To determine whether *Cdkn2aip* was required for spermatogenesis, we used the CRISPR -Cas9 approach to delete *Cdkn2aip* gene (Fig. 2A). The deletion was confirmed as the absence of CDKN2AIP expression and location in the testis of *Cdkn2aip*^*−/−*^ mice measured by western-blot and frozen section (Fig. 2B and C). No obvious difference of viability or growth was identified between *Cdkn2aip*^+*/*+^ and *Cdkn2aip*^*−/−*^ mice. In addition, no obvious change of the structure and weight of testis was discovered in 8-week age *Cdkn2aip*^*−/−*^ mice (Fig. 2D). Our data indicated that the fertility in *Cdkn2aip*^*−/−*^ males were severe declined when co-caged with wildtype female mice (Fig. 2F). Histological examination showed a progressive germ cell and epididymal sperm reduction in *Cdkn2aip*^*−/−*^ mice as they aged (Fig. 2E–G, Additional file 1: Fig. S1 B–E). Taken together, these observations indicated that *Cdkn2aip* deficiency was associated with a dramatic loss of spermatogenic cells.

### ***Cdkn2aip***^−/−^ mice was associated with multiple sperm abnormalities

**Fig. 3:**
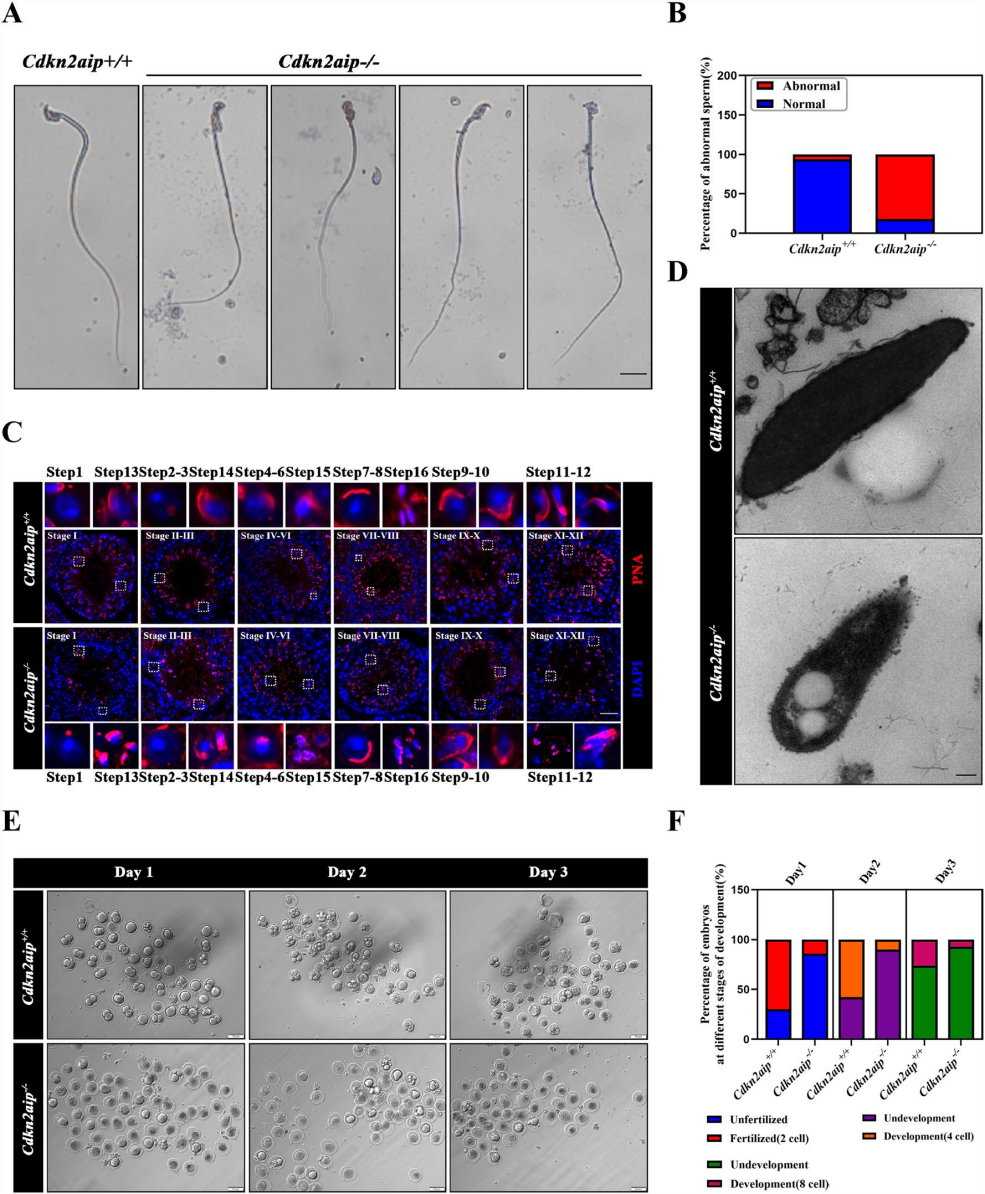
The morphology of sperm head is disrupted in *Cdkn2aip*^*−/−*^ mice. **A** Light microscopy detection of sperm smear after Pap stain. Scale bar, 10 μm. **B** Quantification of spermatozoa with defective head from 8-week-old *Cdkn2aip*^+*/*+^ and *Cdkn2aip*^*−/−*^ mice. Data are presented as mean ± S.D. Student’s *t* test; **P < 0.01. **C** Representative images of spermatozoa from 8-week-old *Cdkn2aip*^+*/*+^ and *Cdkn2aip*^*−/−*^ mice stained for PNA (red) and DAPI (blue). Scale bar, 50 μm. **D** Abnormal sperm head was observed in *Cdkn2aip*^+*/*+^ and *Cdkn2aip*^*−/−*^ mice by transmission EM. Scale bar, 2 μm. **E** The morphology of a wild type sperm fertilizes embryos and *Cdkn2aip*^*−/−*^ sperm fertilizes embryos was examined by light microscopy on days 1 (D1), 2 (D2), and 3 (D3) after fertilization. Scale bar, 100 μm. **F** The percentage of fertilized or developing normal cells in different time periods from the wild type female oocytes after mating with *Cdkn2aip*^+*/*+^ and *Cdkn2aip*^*−/−*^ mice

To further characterize the spermatogenic defects in *Cdkn2aip*^*−/−*^ mice, pap staining of sperm was performed to examine the sperm morphology changes in the epididymis. In contrast to the typical hook-shaped appearance of sperm heads in *Cdkn2aip*^+*/*+^ mice, about 80% sperm heads in *Cdkn2aip*^*−/−*^ mice were amorphous with a smaller and more polygon shape (Fig. 3A and B). Immunofluorescence staining of peanut agglutinin (PNA), a marker of acrosomes, was performed on testicular tissue section. In *Cdkn2aip*^+*/*+^ mice, acrosomes with a typical crescent shape were found on top of the nucleus in the anterior dorsal part of the sperm head as expected. However, the acrosomes of spermatozoa from *Cdkn2aip*^*−/−*^ mice showed multiple defects, including a missing, mis-localized, or fragmented acrosome (Fig. 3C). Transmission EM (TEM) further revealed the presence of abnormal sperm head in *Cdkn2aip*^*−/−*^ mice (Fig. 3D). Next, the fertility assessment was conducted and suggested that most spermatozoa from the *Cdkn2aip*^*−/−*^ mice either failed to fertilize the egg or zygote arrested at early embryo stage (2cell to 4 cell) (Fig. 3E, F). Altogether, these findings demonstrated that abnormal morphology of *Cdkn2aip* spermatids occurred when spermatids start to elongate (Additional file 2).

### Spermatogenesis failure is caused by failed histone replacement in sperm ***Cdkn2aip***^***−/−***^ mice

**Fig. 4:**
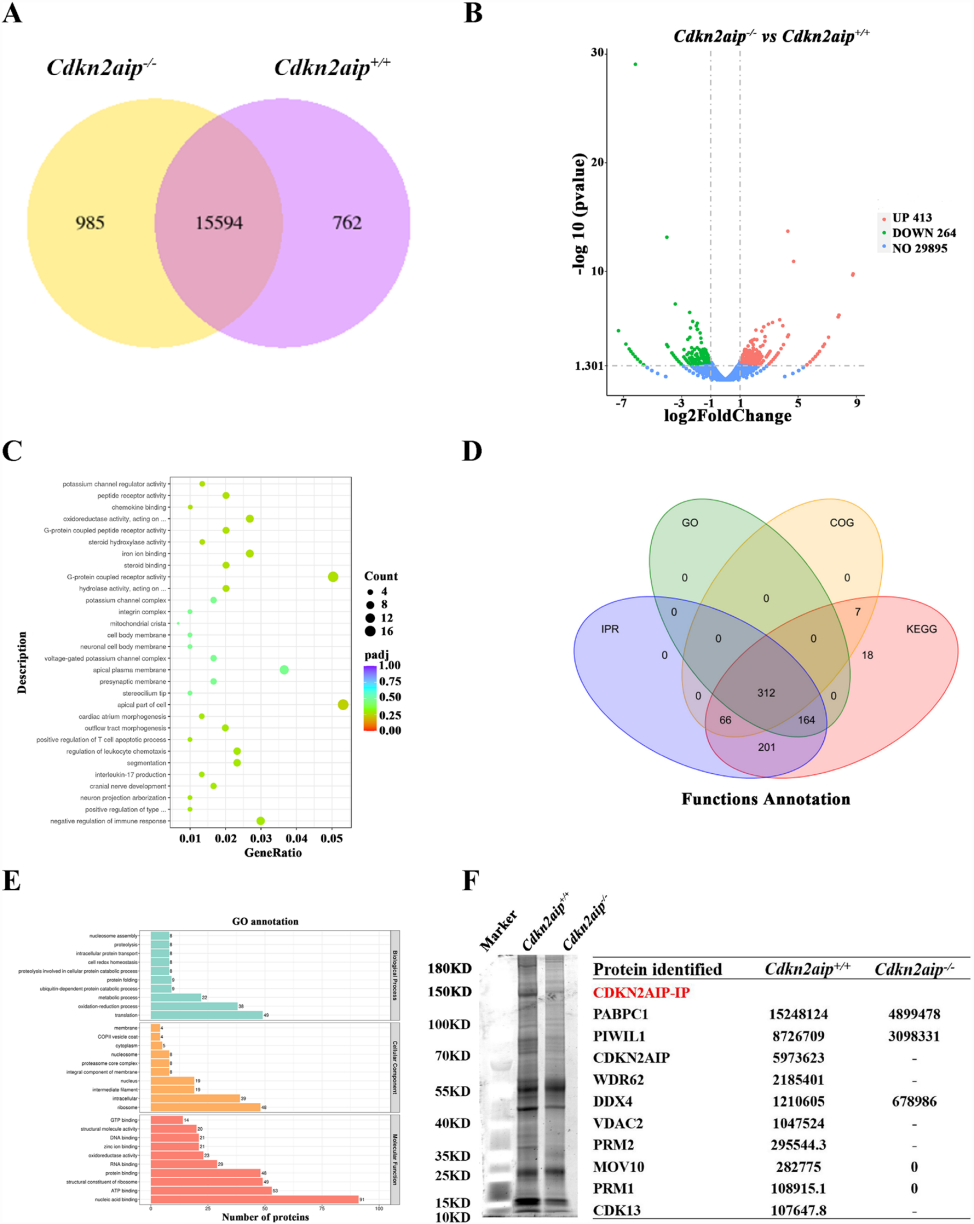
Identification of the Related genes and proteins affected by *Cdkn2aip* mutation using RNA-Seq and IP-MS. **A** Venn diagram showing the number of unique transcript isoforms detected in *Cdkn2aip*^+*/*+^ (762) and *Cdkn2aip*^*−/−*^ (985) testes. **B** RNA-Seq analysis of differentially expressed genes from *Cdkn2aip*^+*/*+^ and *Cdkn2aip*^*−/−*^ testes. **C** The GO enrichment ratio for identified genes was performed on RNA Seq data from *Cdkn2aip*^+*/*+^ and *Cdkn2aip*^*−/−*^ testis. **E** Results of function annotation and gene ontology (GO) term enrichment analyses of CDKN2AIP-interacting proteins by IP-MS. **F** A representative gel image showing bands proteins immunoprecipitated by CDKN2AIP antibody in the *Cdkn2aip*^+*/*+^ and *Cdkn2aip*^*−/−*^ testis (left), and the list of partial differential proteins form the *Cdkn2aip*^+*/*+^ and *Cdkn2aip*^*−/−*^ testis by IP-MS (right)

**Fig. 5:**
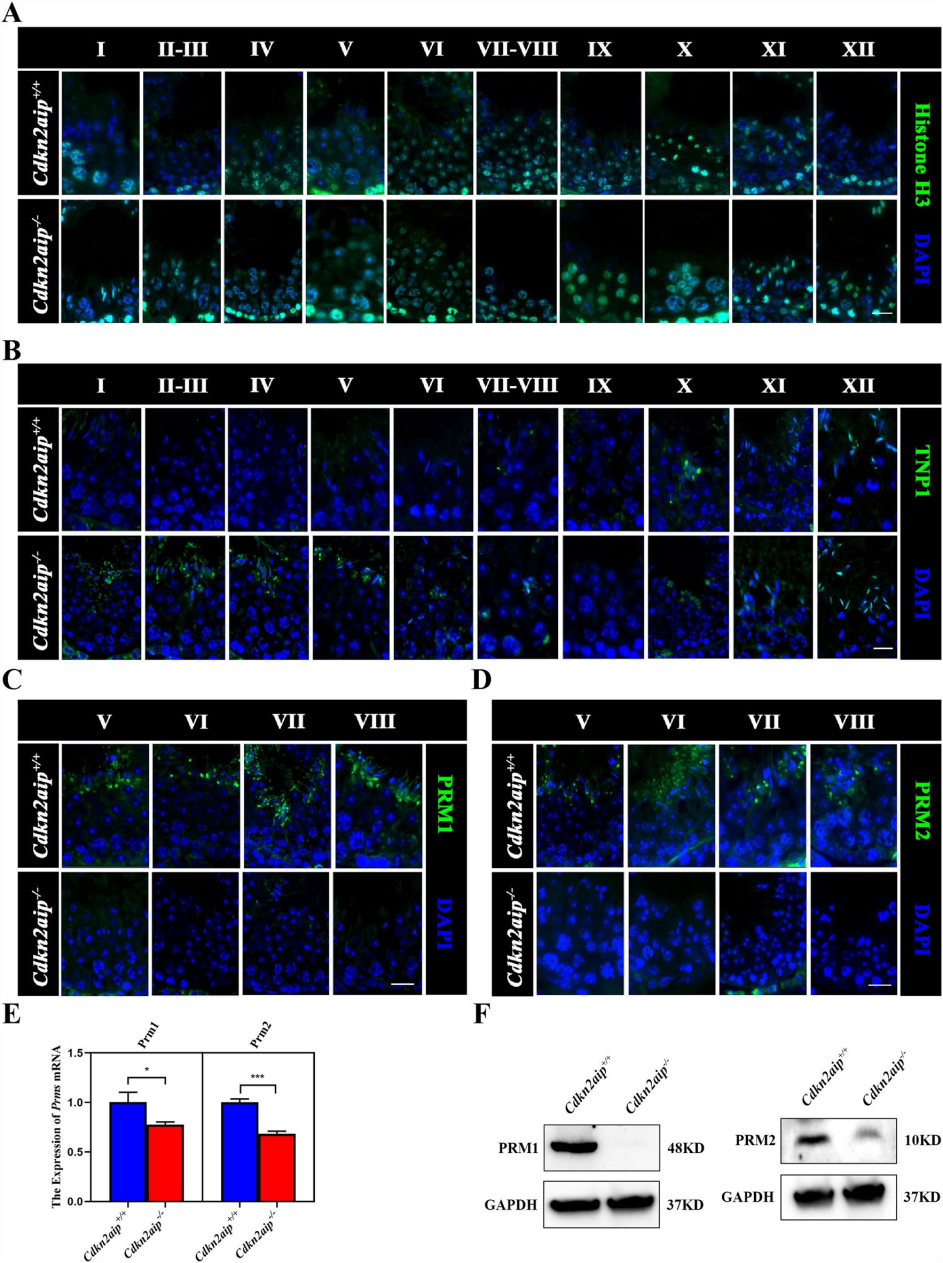
Disruption of the affect protamine replacement in *Cdkn2aip-/-* mice. **A** Immunofluorescence staining of histone H3 in seminiferous tubules of *Cdkn2aip*^+*/*+^ and *Cdkn2aip*^*−/−*^ testes. histone H3(green) and DAPI (blue). Scale bar, 50 μm. **B** Immunofluorescence staining of TNP1 in seminiferous tubules of *Cdkn2aip*^+*/*+^ and *Cdkn2aip*^*−/−*^ testes. TNP1 (green) and DAPI (blue). Scale bar, 50 μm. **C** and **D** Immunofluorescence staining of PRM1 and PRM2 in seminiferous tubules of *Cdkn2aip*^+*/*+^ and *Cdkn2aip*^*−/−*^ testes. PRMs(green) and DAPI (blue). Scale bar, 50 μm. **E** qPCR analyses showing significantly reduced levels of Prm1 and Prm2 in 8-week-old *Cdkn2aip*^+*/*+^ and *Cdkn2aip*^*−/−*^ testis. Data are presented as Data are presented as mean ± S.D. Student’s *t* test; *P < 0.05, ***P < 0.001. **F** Quantification of relative protein level of PRM1 and PRM2 by Western blotting in *Cdkn2aip*^+*/*+^ and *Cdkn2aip*^*−/−*^ testis. GAPDH is serves as a loading control

To explore the cause of spermatogenesis failure, we further performed RNA-Seq analyses using *Cdkn2aip*^+*/*+^ and *Cdkn2aip*^*−/−*^ testis obtained from mice at the age of 35 days. RNA-Seq analyses revealed that a total of 985 unique expressed genes were detected exclusively in the *Cdkn2aip*^*−/−*^ testes, as compared to only 762 unique expressed genes in the *Cdkn2aip*^+*/*+^ testes (Fig. 4A). Further analyses identified 413 upregulated and 264 downregulated genes in *Cdkn2aip*^*−/−*^ testes as compared to *Cdkn2aip*^+*/*+^, and the expressions of protamines (PRMs) and transition proteins (TNPs) were significantly decreased (Fig. 4B). GO functional enrichment analysis showed that *Cdkn2aip* was mainly involved in the apical part of cells (Fig. 4C). To determine which protein might be involved in sperm deformation in testis isolated from *Cdkn2aip*^+*/*+^ and *Cdkn2aip*^*−/−*^ mice, we performed immunoprecipitation-mass spectrometry (IP-MS) assays using a well-validated CDKN2AIP antibody. A total of 312 proteins were jointly annotated by GO, KEGG, COG and IPR (Fig. 4D). GO enrichment analyses revealed that the function of the proteins identified mainly involved in nucleic acid-binding/processing biological processes (Fig. 4E). Most notably, we identified that the expression of PRMs was affected in *Cdkn2aip*^−/−^ mice (Fig. 4F). In addition, results of immunofluorescence showed normal histone H3 localization was occurred in *Cdkn2aip*^+*/*+^ and *Cdkn2aip*^*−/−*^ during spermatogenesis (Fig. 5A). But the replaced of TNP2 significantly delay in the *Cdkn2aip*^*−/−*^ testes (Fig. 5B). The staining of PRMs was significantly reduced in *Cdkn2aip*^*−/−*^ mice (Fig. 5C and D). qPCR and Western blot also showed that the expression of PRMs was significantly reduced in the testes of *Cdkn2aip*^*−/−*^ mice (Fig. 5E and F). Collectively, the cause of sperm abnormality in *Cdkn2aip*^*−/−*^ mice is a failure of spermiogenesis (Additional file 5: Fig. S5A).

### CDKN2AIP is involved in the post-transcriptional regulation of SUN1 expression to maintained the development of the germ cell

**Fig. 6:**
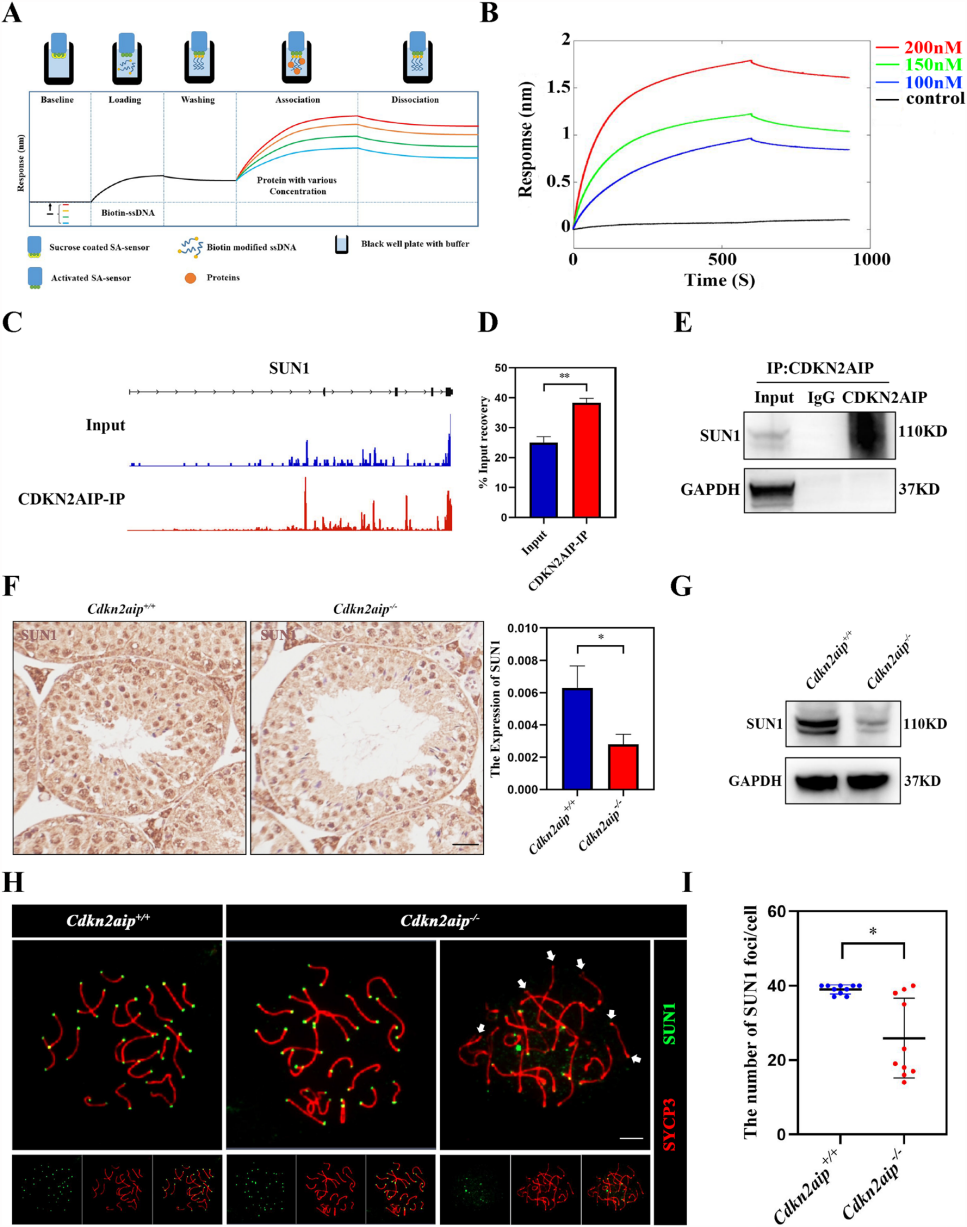
Disruption of the SUN1 expression and location in *Cdkn2aip*^*−/−*^ mice. **A** Schematic representation of Bio-layer Interferometry (BLI). **B** Association and dissociation curves of CDKN2AIP protein combining with the ssDNA in a concentration range between 100 mM(blue), 150 mM(green), 200 mM(red). **C** A representative mRNA assembly output showing RIP-Seq reads for Sun1 identified from the RIP products using the CDKN2AIP antibody and Control. The red peak is the anti-CDKN2AIP bond and the bule peak is the control group. **D** qPCR analyses of level of CDKN2AIP-bound Sun1 mRNA in RIP products and Control. Data are presented as mean ± S.D. Student’s *t* test; **P < 0.01. **E** Validation of interactions between CDKN2AIP and SUN1 in protein level by co-immunoprecipitation assays, in which antibodies specific for CDKN2AIP was used for immunoprecipitation (IP) followed by western blot using anti-SUN1 antibody. IgG was used as a control. **F** Localization of SUN1 in the *Cdkn2aip*^+*/*+^ and *Cdkn2aip*^*−/−*^ testis detected by immunohistochemistry. SUN1 is stained brown. Scale bar, 50 μm; Quantification of SUN1-positive signal in five horizons per group. Data are presented as mean ± S.D. Student’s *t* test; *P < 0.05. **G** Western blot analyses the expression of SUN1 in *Cdkn2aip*^+*/*+^ and *Cdkn2aip*^*−/−*^ testis. GAPDH is serves as a loading control. **H** Immunostaining of SUN1(green) and SYCP3(red) on chromosome spreads of spermatocytes from P35 *Cdkn2aip*^+*/*+^ and *Cdkn2aip*^*−/−*^ testes, n = 3 mice for each group. Scale bars, 10 μm. **I** Statistical analysis of the number of SUN1 positive signals in 10 pachytene spermatocytes from P35 *Cdkn2aip*^+*/*+^ and *Cdkn2aip*^*−/−*^ testes. n = 3 mice for each group, and 10 pachytene spermatocytes were counted for each mouse. Data are presented as mean ± SD, **P < *0.01* by two-tailed Student’s-test

The full-length CDKN2AIP protein was purified and used to examine the interaction between protein and ssDNA by biolayer interferometry (BLI) technique. The BLI result showed high binding affinity of CDKN2AIP to single-stranded DNA (Fig. 6A and B), but we failed in the experiment of chip and seq. Further, we performed RNA-immunoprecipitation followed by next-gene sequencing (RIP-Seq) assays and the RIP-Seq data identified a total of 2829 transcripts that were significantly enriched in the CDKN2AIP immunoprecipitants. Among all RNAs, we discovered that the Sun1, which involved in the nuclear shaping, was abundantly enriched in the immunoprecipitation (Fig. 6C). qPCR of the RIP products verified CDKN2AIP antibody is enriched to more abundant Sun1 than Input (Fig. 6D). To further verify whether CDKN2AIP protein interacted with SUN1 in the testes of mice, we performed co-immunoprecipitation with testis lysates from *Cdkn2aip*^+*/*+^. Western blotting detected SUN1 in lysates immunoprecipitated by the anti-CDKN2AIP antibody but not in IgG-immunoprecipitated lysates (Fig. 6E). Immunohistochemistry and Western blot showed that the expression of SUN1 was reduced in testis of adult *Cdkn2aip*^*−/−*^ mice compared with *Cdkn2aip*^+*/*+^ (Fig. 6F, G). We performed chromosome spread immunofluorescence staining of SUN1 further and found out that the SUN1 foci were partial and unevenly distributed in each cell in *Cdkn2aip*^*−/−*^ mice (Fig. 6H, I). Taken together, our data suggested that CDKN2AIP is involved in the post-transcriptional regulation of SUN1 expression to maintained the development of the germ cell (see Additional file 6).

### Disruption of *Cdkn2aip* impaired meiosis progression and promoted apoptosis of germ cell

**Fig. 7:**
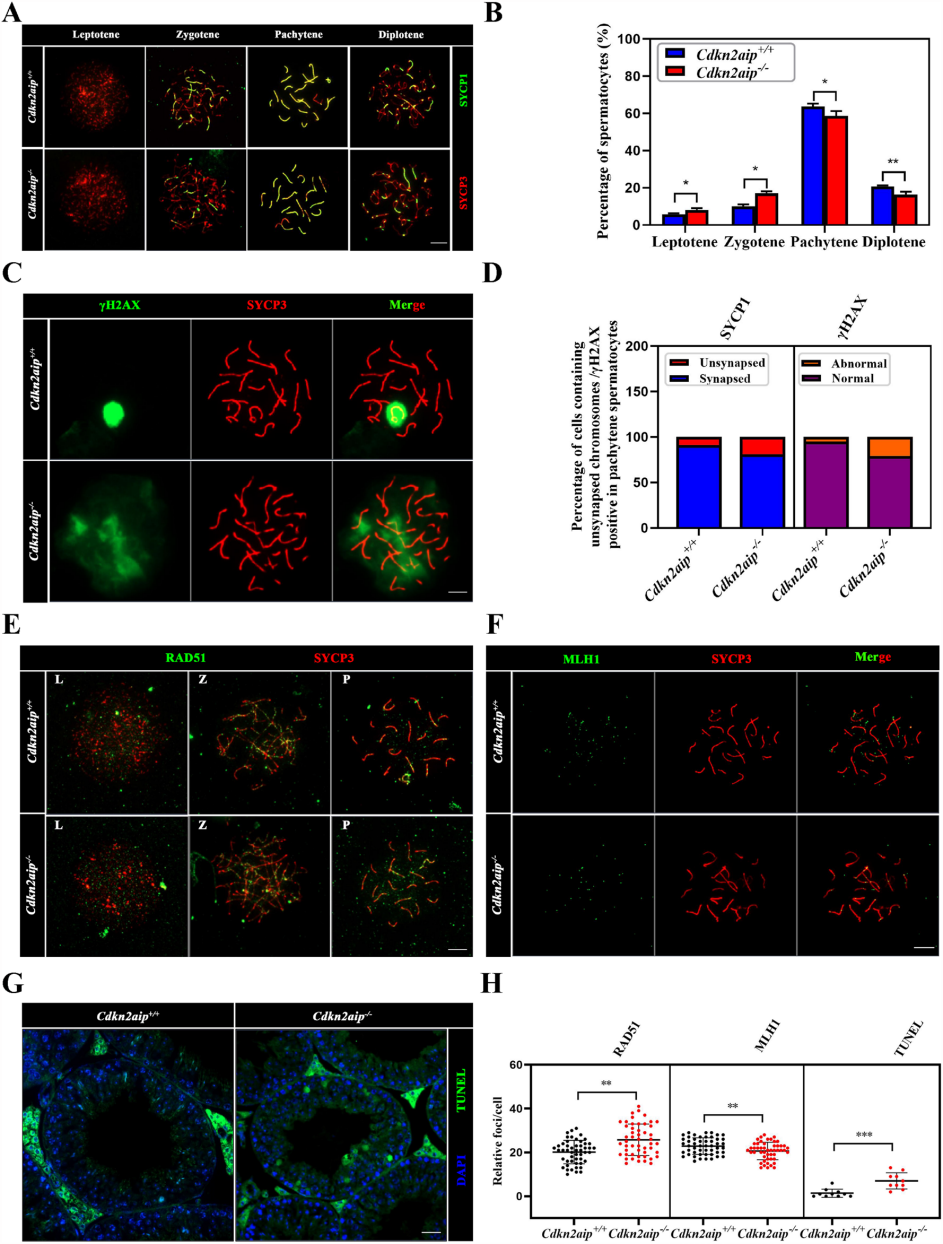
Uncompleted DNA double-strand breaks repair and synapsis in *Cdkn2aip*^*−/−*^ spermatocytes. **A** Immunostaining of SYCP1(green) and SYCP3(red) on chromosome spreads of spermatocytes from P35 *Cdkn2aip*^+*/*+^ and *Cdkn2aip*^*−/−*^ testes, n = 3 mice for each group. Scale bars, 10 μm. **B** Frequency of meiotic prophase I stages. n = 3 mice for each group, and 50 spermatocytes from each mouse were examined. Data are presented as mean ± SD, *P < 0.05 **P < *0.01* by two-tailed Student’s-test. **C** Immunostaining of γH2AX (green) and SYCP3(red) on chromosome spreads of spermatocytes from P35 *Cdkn2aip*^+*/*+^ and *Cdkn2aip*^*−/−*^ testes, n = 3 mice for each group. Scale bars, 10 μm. **D** Statistical results of (**C**). Data are presented as average percentage, n = 3 mice for each group, and 50 pachytene spermatocytes were counted for each mouse. **E** Immunostaining of RAD51 (green) and SYCP3(red) on chromosome spreads of spermatocytes from P35 *Cdkn2aip*^+*/*+^ and *Cdkn2aip*^*−/−*^ testes, n = 3 mice for each group. Scale bars, 10 μm. **F** Immunostaining of MLH1 (green) and SYCP3(red) on chromosome spreads of spermatocytes from P35 *Cdkn2aip*^+*/*+^ and *Cdkn2aip*^*−/−*^ testes, n = 3 mice for each group. Scale bars, 10 μm. **G** TUNEL assays on *Cdkn2aip*^+*/*+^ and *Cdkn2aip*.^*−/−*^ testes. Arrows point to apoptotic cells stained in green. Scale bar, 50 μm. **H** Statistical results of (**E**–**G**). 50 spermatocytes of pachytene stage were counted for RAD51 and MLH1 foci. 10 seminiferous tubules were counted in each group. Data are presented as mean ± S.D. Student’s *t* test; **P < 0.01 ***P < 0.001

To further characterize the defects of *Cdkn2aip*^*−/−*^ testis, germ cell spreads were prepared and stained for key meiosis-associated proteins. The location of SYCP1, a key protein for maintaining chromosome integrity, was disrupted in 19% of the *Cdkn2aip*^*−/−*^ spermatocytes (Fig. 7A and D), suggesting that meiotic synapsis was incomplete and partially impaired. Spermatocytes were counted at different stages of meiosis prophase I, and the results showed that the percentage of diplotene spermatocytes was decreased, while the percentage of leptotene and zygotene increased in *Cdkn2aip*^*−/−*^ mice (Fig. 7B). Furthermore, we discovered that the HORMAD1, which normally only presents on the well-compacted sex chromosomes, also presented on autosomal chromosomes of *Cdkn2aip*^*−/−*^ pachytene cells (Additional file 3: Fig. S3A and B). The DNA damage repair activation and DDR kinetics was determined by the phosphorylation of H2AX at Ser 139 (γ-H2AX) foci formation, which represented DSB sites in the nucleus. γ-H2AX is the most sensitive marker that can be used to examine the DNA damage produced and the subsequent repair of the DNA lesion. In wildtype pachytene spermatocytes, since the repair of autosomes has been completed, γH2AX only exists in the incomplete repaired sex chromosome region. However, by comparing the timing of γH2AX disappearance of autosomes in pachytene spermatocytes, it is proposed that DSB repair delayed in *Cdkn2aip*^*−/−*^ mice. (Fig. 7C, D and Additional file 3: S3C, D). However, the DSB repair-associated recombinase RAD51 was maintained at higher level while the expression of MLH1 was declined in *Cdkn2aip*^*−/−*^ pachytene spermatocytes compared with *Cdkn2aip*^+*/*+^ (Fig. 7.E, F, H). Terminal deoxynucleotidyl transferase dUTP nick end labeling (TUNEL) analysis exhibited a significant increase in apoptosis within mutant seminiferous tubules (Fig. 7G, H). Collectively, these results suggested that knocking out of *Cdkn2aip* impaired DSB repair ability and leads to genomic instability during meiosis.

### Reduction of *Cdkn2aip* expression was associated with cell cycle arrest and increased apoptosis in TM4 cells

**Fig. 8:**
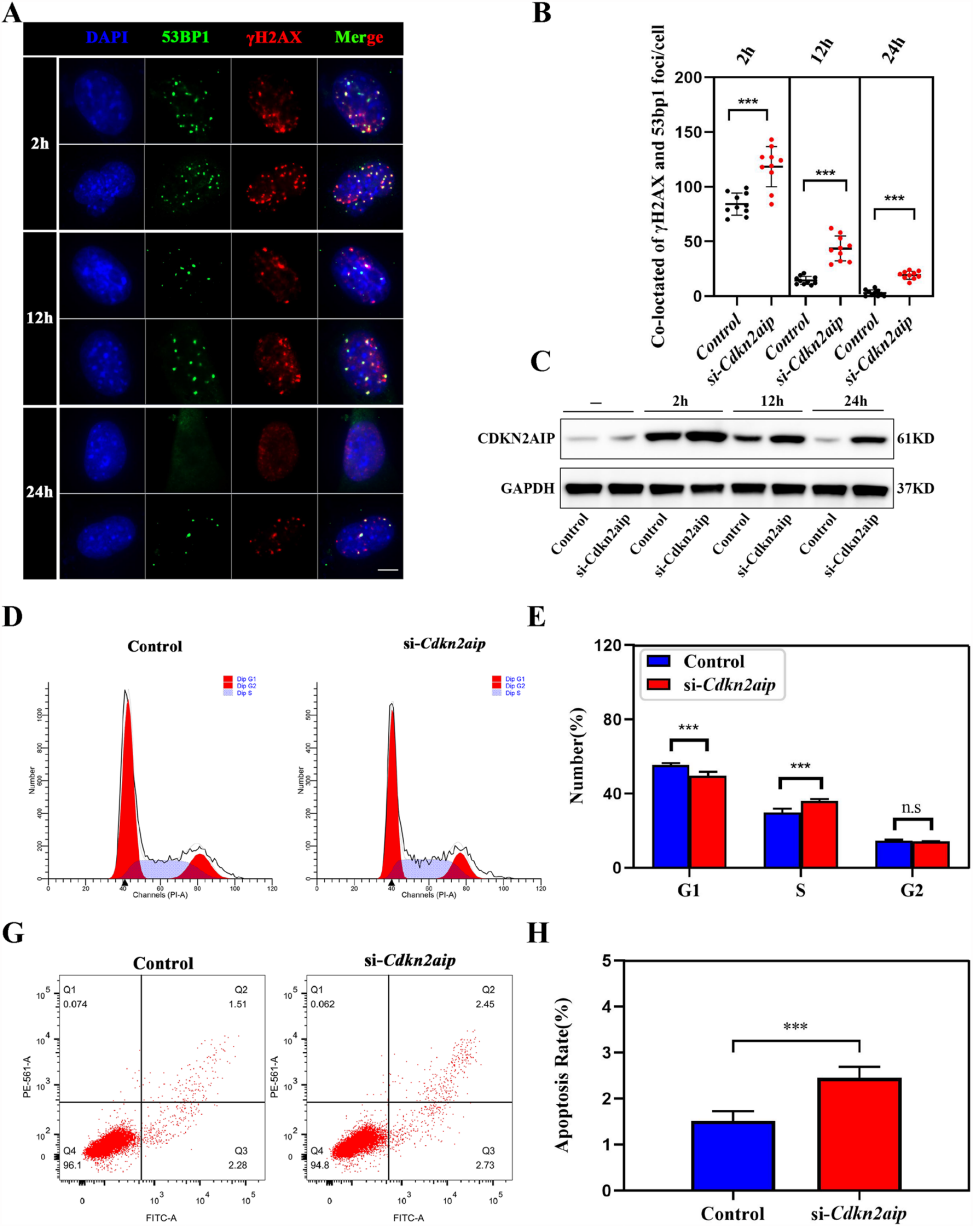
Reduction of *Cdkn2aip* impairs DNA repair, prolong cell cycle and promote apoptosis in TM4. **A** *Cdkn2aip* knockdown TM4 cell by exposure to 5 Gy γ-irradiation show more γH2AX foci after 12 h and 24 h recovery compared with control by immunofluorescence. γH2AX (red), 53BP1(green) and DAPI (blue). Scale bar, 5 μm. **B** Quantitative results of co-positive signal of γH2AX and 53BP1. n = 10 cells were counted. Data are presented as mean ± S.D. Student’s *t* test; **P < 0.01. **C** Western blot analysis the expression of CDKN2AIP in *Cdkn2aip* knockdown TM4 cell and control treated by 5 Gy γ-irradiation, GAPDH is serves as a loading control. **D**–**H** Cell cycle and apoptosis analysis of Control and *Cdkn2aip* knockdown TM4 cells by flow cytometry. Data are presented as mean ± S.D. Student’s *t* test; ***P < 0.001

To better understand the mechanism of how *Cdkn2aip* affect cell cycle, its expression was knocked-down in TM4 cells by 5G-γ-irradiation. We then measured the expression and locating of γH2AX and 53BP1, commonly used DNA damage response markers, in Control and *Cdkn2aip* knockdown TM4 cells with western blot and immunofluorescence. After recovery for 12 h, higher γH2AX levels accumulated in *Cdkn2aip* knockdown TM4 cells compared with control. At 24 h, the repair was complete in the control group while the γH2AX foci was still present in the *Cdkn2aip* knockdown group (Fig. 8A–C). The co-located 53BP1with γH2AX also persisted longer at higher level at 12 h and 24 h in *Cdkn2aip* knockdown TM4 cells than in the controls after exposure to γ-irradiation (Fig. 8A). The localization of γH2AX and 53BP1 in pachytene spermatocytes of mice was significantly abnormal (Additional file 3: Fig. S3C–F). Analysis of cell cycle and apoptosis markers suggested extended S phase and more frequent apoptosis were observed in *Cdkn2aip* knockdown TM4 cells compared to the control (Fig. 8D–F). These data further indicated that reduction of *Cdkn2aip* impaired DSB repair and increased cell apoptosis.

## Discussion

Spermatocyte development and spermiogenesis are two key meiosis events during mammalian spermatogenesis. In this study, we identified CDKN2AIP as a testis-enriched protein in mice and almost no expression in other tissues. Hence, we generated *Cdkn2aip*-KO mice using CRISPR/Cas9, which was confirmed by Sanger sequencing and western blot. Our result shows that CDKN2AIP localized at the spermatocytes to round spermatozoa during meiosis. The germ cell reduction from *Cdkn2aip*^*−/−*^ mice showed partial arrest at meiosis with impaired synapsis and DSB repair, CDKN2AIP targeted SUN1 to maintain normal process of meiosis prophase I. In addition, the loss of *Cdkn2aip* led to multiple sperm head defects resulting from failure of protamine replacement. We report, for the first time, that *Cdkn2aip* is indispensable for spermiogenesis and germ cell development.

*Cdkn2aip*^*−/−*^ increased spermatocyte apoptosis accompanied by abnormal DSB repair, synapsis and crossover formation. Persistent γH2AX foci provides molecular marker of DNA damage and can accelerate aging [21]. Moreover, impaired DSB repair and accumulation of DNA lesions contribute to age-associated rise of genomic instability and age-related diseases [22–24]. CDKN2AIP can bind to the tumor suppressor gene p53 to regulate the transcription of the gene encoding the cell cycle inhibitor p21-CDKN1A [25]. Once *Cdkn2aip* was knocked out, cells failed to properly induce p21-CDKN1A expression, which might result in augmented DNA damage with increased p53-dependent apoptosis. Thus, it is most likely that the cell death in the tubule lumens of the testes we observed in our study was associated with the loss of *Cdkn2aip* associated cell cycle regulatory function. Our data showed that reduced expression of CDKN2AIP induced germ cell apoptosis which contribute to age dependent infertility. The apoptosis of the cells appears to be due to the cell cycle and survival regulatory functions of *Cdkn2aip*. In addition, these observations suggest CDKN2AIP may be involved in processing of post-meiotic DSB repair.

The spermiogenesis is characterized by nuclear restructuring, a highly complex process which ensures the differentiation from immature haploid male germ cells into mature, fertilization competent spermatozoa [26]. A most prominent feature in this process is the re-shaping of the sperm nucleus from spherical to elongated [27]. The RIP-Seq and IP-MS data of our study revealed that many proteins, such as SUN1, participated in apical partial condensation, and could be affected by expression CDKN2AIP, which suggested an unrecognized function of CDKN2AIP in regulating sperm head chromatin condensation. It has been known that SUN1 has a major role in shaping nuclear during sperm head formation and maintaining normal nuclear morphology. It physically connects the nucleus with the peripheral cytoskeleton and is critically involved in a variety of dynamic processes, such as nuclear anchorage, movement and positioning and meiotic chromosome dynamics. Anchorage and positioning of the cell nucleus play an important role during diverse developmental processes such as fertilization, cell migration, and establishment of polarity [28]. Previously study reported that *Sun1* provides a structural bridge that connects the nucleus to cytoplasmic actin and is involved in nuclear anchorage [29]. Truncation of the cryptic N-terminal chromatin-binding domain of Sun1 induces dramatic separation of the inner from the outer nuclear membrane and results in deformations of nuclear morphology, which are also observed using a *Sun1* RNAi construct [30]. Here in this study, we not only showed that the SUN1 function was affected by CDKN2AIP, but also shed light upon a better understanding of this interesting pathway.

The SUN1 was the first identified protein connecting structures between telomeres and the NE during meiotic chromosome pairing and synapsis [11, 31, 32]. Mice deficiency of SUN1 showed failure of chromosome recombination and synapsis [7]. Here, we found that the *Cdkn2aip*^*−/−*^ mice displayed a similar incomplete reorganization and synapsis, which may be associated with the decreased expression of SUN1. It is important to point out that unlike *Sun1*^*−/−*^ mice, the spermatocyte of *Cdkn2aip*^*−/−*^ mice was partially reserved at an early age. This might be explained by that the expression of SUN1 was partially reserved in *Cdkn2aip*^*−/−*^ mice at an early age.

During the differentiation of round spermatids into spermatozoa, one major event is the tight compaction of sperm nucleus which achieved through the replacement of most histones TNPs and subsequently with PRMs [33, 34]. Protamines are major DNA-binding proteins in nucleus of sperm and package the DNA in a volume less than 5% of a somatic cell nucleus [35]. Many mammals have one type of protamine while a few species, including humans and mice, have two [36, 37]. A decrease in the amount of either protamine disrupts nuclear formation and normal sperm function [38, 39]. Our studies suggested that *Cdkn2aip* participated in spermiogenesis by regulating the expression of specific target genes such as *Prm1* and *Prm2*, which are required for histone replacement during the final stages of sperm chromatin condensation and maturation. The decreased of protamines expression in *Cdkn2aip*^*−/−*^ mice may be a cause of low fertility with apparently decreased sperm production. Abnormal protamine expression may be indicative of a general abnormality of spermatogenesis. It has been known that abnormal protamine expression leads to apoptotic process and results in severely diminished semen quality. T Shiozawa et al. discovered the spermatozoa with morphologic anomalies contained less protamines and more histones than normal spermatozoa [40]. In addition to the above potential functions, it has also been proposed that protamines is a key regulatory factor of sperm nuclear assembly and participates in the fertilization process [41]. Also, protamines themselves could confer an epigenetic mark on some regions of the sperm genome, affecting its reactivation upon fertilization [42]. After fertilization, the highly packaged nucleoprotamine sperm genome must be decondensed [43]. The chromatin changes and unpacking after fertilization potentially relevant to the function of protamines are reviewed elsewhere. These observations may help to explain why decreased PRMs expression in *Cdkn2aip*^*−/−*^ were associated with morphological abnormalities, initiation of apoptotic pathway and decreasing sperm motility and impaired fertilization.

## Conclusions

In summary, our study found that *Cdkn2aip*^*−/−*^ mice exhibited multiple sperm head defects accompanied by the failure of chromosomal synapsis and DNA repair near telomeres, indicating a novel and critical function of *Cdkn2aip* in the process of spermiogenesis and germ cell development.

## Supplementary Information

**Additional file 1: Figure S1.** Targeted knock-out of the *Cdkn2aip* Gene resulted in age-dependent infertility in male mice. (A) qPCR analyses of *Cdkn2aip* mRNA levels in multiple organs in mice, β-actin is serves as a housekeeping gene. (B) Litter sizes from wild type females mated with either* Cdkn2aip*^*+/+*^ and *Cdkn2aip*^*−/−*^ mice in 14-week-old (n = 6/group). Data are presented as mean ±S.D. Student’s t test; ***P < 0.001. (C) Testicular sections of 14-week-old* Cdkn2aip*^*+/+*^ and* Cdkn2aip*^*-/-*^ were stained with H&E. Scale bar, 50 μm. (D) CDKN2AIP full-length protein was purified and verified by SDS electrophoresis. (E) Statistical results of the number of seminiferous tubules with less or without germ cell. n=3 mice for each group, and 100 tubules were counted for each mouse. (F) Relative number of epididymal sperm number of 14-week-old *Cdkn2aip* ^*+/+*^ and *Cdkn2aip*^*-/-*^ mice (n=6). Data are presented as mean ±S.D. Student’s t test; ***P <0.001.

**Additional file 2: Figure S2.** *Cdkn2aip*^*-/-*^ mice exhibit normal spermatogonia and Sertoli cells development. (A) Frozen section staining of SOX9 (green) in testicular tissue from P56 *Cdkn2aip* ^*+/+*^ and *Cdkn2aip*^*-/-*^ mice. Scale bar, 50μm. (B) Statistical results of (A). Data are presented as average percentage, n=3 mice for each group, and 50 tubules were counted for each mouse. (C) Frozen section staining of PLZF (red) in testicular tissue from P56 *Cdkn2aip* ^*+/+*^ and *Cdkn2aip*^*-/-*^ mice. Scale bar, 50μm. (D) Statistical results of (C). Data are presented as average percentage, n=3 mice for each group, and 50 tubules were counted for each mouse. (E) Frozen section staining of STRA8 (green) in testicular tissue from P56 *Cdkn2aip* ^*+/+*^ and *Cdkn2aip*^*-/-*^ mice. Scale bar, 50μm. (F) Statistical results of (E). Data are presented as average percentage, n=3 mice for each group, and 50 tubules were counted for each mouse.

**Additional file 3: Figure S3.** *Cdkn2aip*^*-/-*^ mice exhibit abnormal autosomal synapsis at meiosis prophase I. (A) Immunostaining of SYCP3(red) and HORMAD1(green) in *Cdkn2aip* ^*+/+*^ and *Cdkn2aip*^*-/-*^ pachytene spermatocytes, n=3 mice for each group Scale bars, 10μm. (B) Statistical results of (A). Data are presented as average percentage, n=3 mice for each group, and 50 spermatocyte were counted for each mouse. (C) Frozen section staining of γH2AX (green) and DIPA (blue) in testicular tissue from P56 *Cdkn2aip* ^*+/+*^ and *Cdkn2aip*^*-/-*^ mice. Scale bar, 50μm. (D) Statistical results of abnormal γH2AX signals/tubule. n=3 mice for each group, and 20 tubules were counted for each mouse. Data are presented as mean±SD, ^***^P<0.001 by two-tailed Student’s-test. (E) Immunostaining of 53BP1 (green) and SYCP3(red) and on chromosome spreads of spermatocytes from P35 *Cdkn2aip* ^*+/+*^ and *Cdkn2aip*^*-/-*^ mice testes, n=3 mice for each group. Scale bars, 10μm. (F) Statistical analysis of abnormal location percentage of 53BP1 in pachytene spermatocytes. Data are presented as average percentage, n=3 mice for each group, and 100 pachytene spermatocytes were counted for each mouse.

**Additional file 4: Figure S4.** Principle and data analysis process of RNA Seq and IP-MS. (A) The details of experimental operation and data analysis of RNA seq. (B) The details of experimental operation and data analysis of IP-MS.

**Additional file 5:** **Figure S5.** Schematic diagram showing the proposed model of CDKN2AIP function during spermatogenesis in mice

**Additional file 6: Table S1.** Primer sequences are used in this study.

## Data Availability

All the data is contained in the manuscript.

## Abbreviations

CDKN2AIP: CDKN2A interacting protein
DSB: DNA double strand breaks
DDR: DNA damage repair
SYCP3: Synaptonemal Complex Protein 3
HORMAD1: HORMA domain containing 1
Trp53BP1: Transformation related protein 53 binding protein 1
NE: Inner nuclear membrane
