# Supplementary figures and images for: CDKN2AIP is critical for spermiogenesis and germ cell development
Source: Cell Biosci. 2022 Aug 21;12:136. doi: 10.1186/s13578-022-00861-z (PMC9394077; doi:10.1186/s13578-022-00861-z)

**A**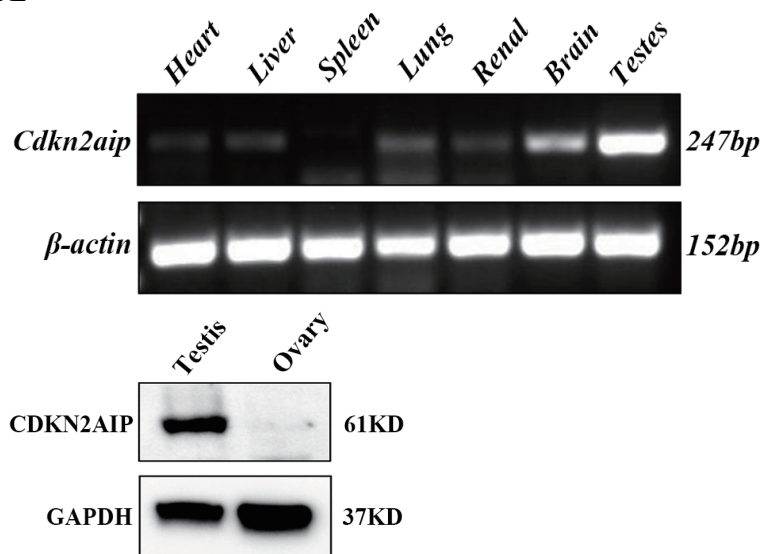**B**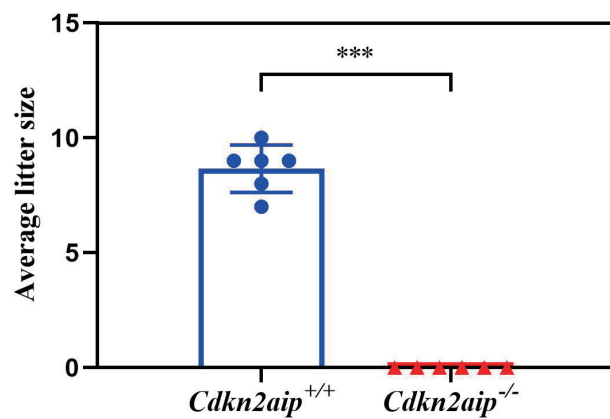**C**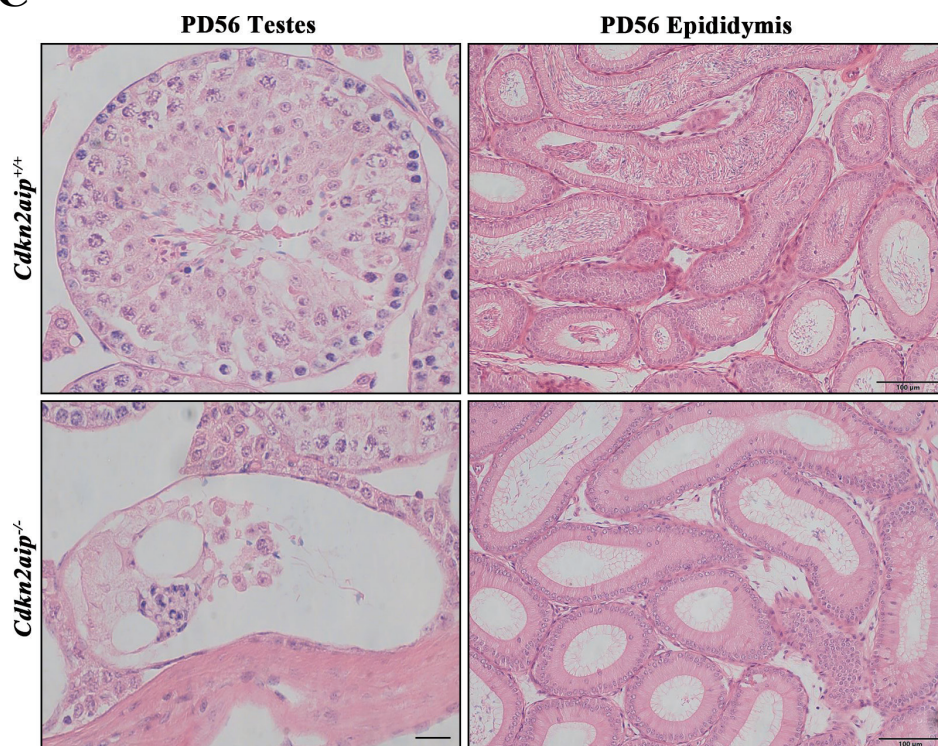**D**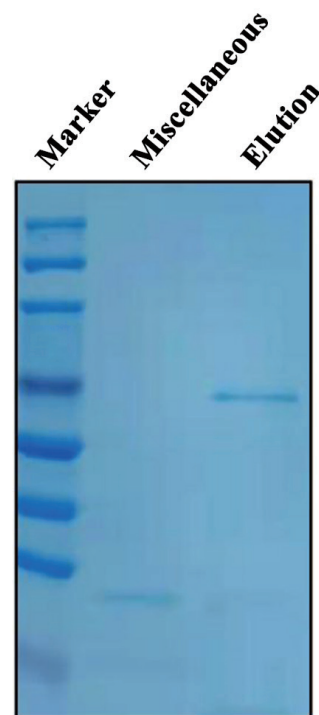**E**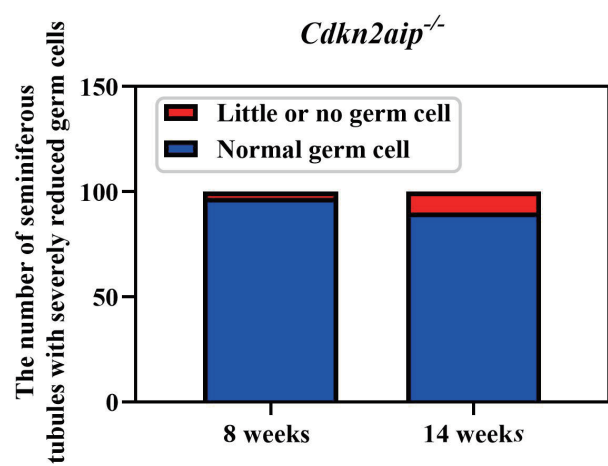**F**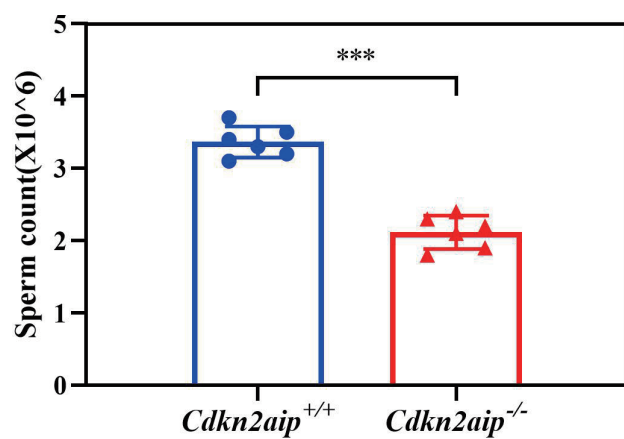

Supplement: Supplementary file 1 — Additional file 1: Figure S1. Targeted knock-out of the Cdkn2aip Gene resulted in age-dependent infertility in male mice. (A) qPCR analyses of Cdkn2aip mRNA levels in multiple organs in mice, β-actin is serves as a housekeeping gene. (B) Litter sizes from wild type females mated with either Cdkn2aip+/+ and Cdkn2aip−/− mice in 14-week-old (n = 6/group). Data are presented as mean ±S.D. Student’s t test; ***P < 0.001. (C) Testicular sections of 14-week-old Cdkn2aip+/+ and Cdkn2aip-/- were stained with H&E. Scale bar, 50 μm. (D) CDKN2AIP full-length protein was purified and verified by SDS electrophoresis. (E) Statistical results of the number of seminiferous tubules with less or without germ cell. n=3 mice for each group, and 100 tubules were counted for each mouse. (F) Relative number of epididymal sperm number of 14-week-old Cdkn2aip +/+ and Cdkn2aip-/- mice (n=6). Data are presented as mean ±S.D. Student’s t test; ***P <0.001. [file 13578_2022_861_MOESM1_ESM.pdf]

A

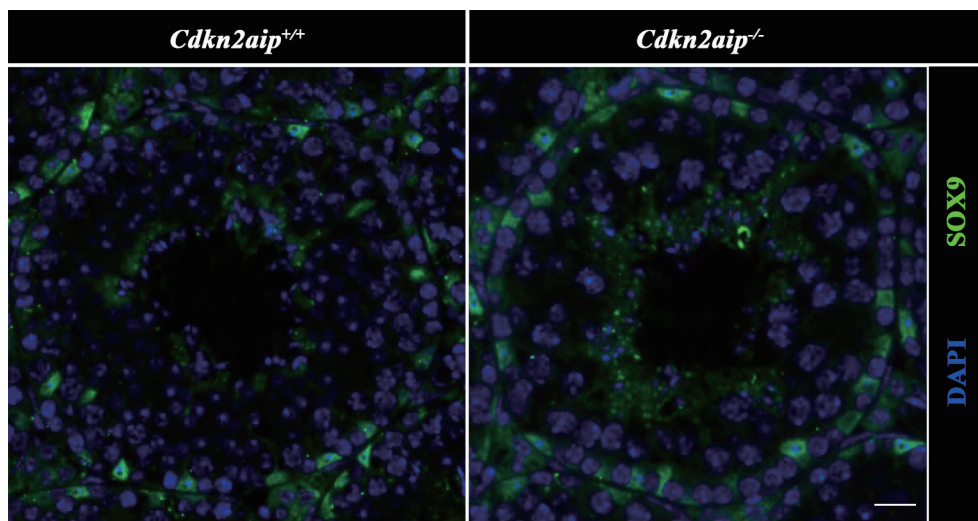

B

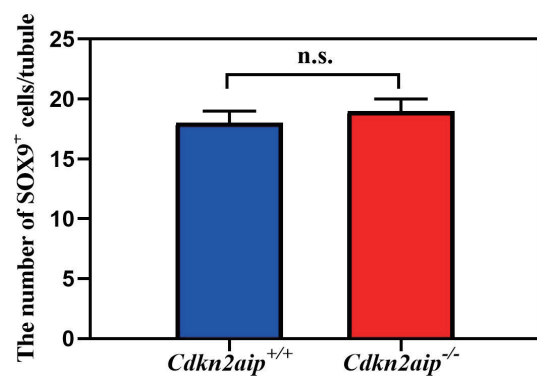

C

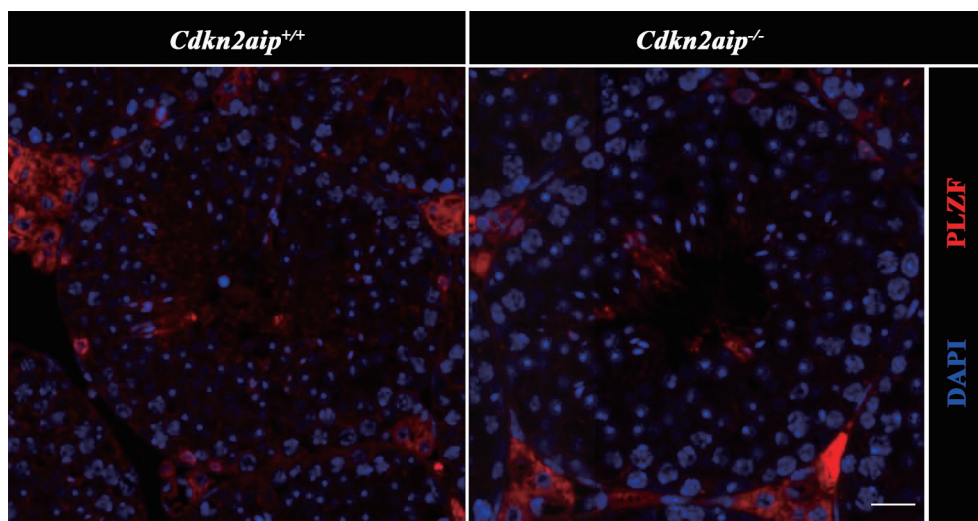

D

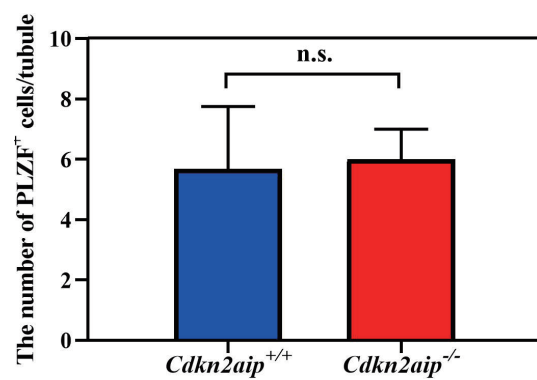

E

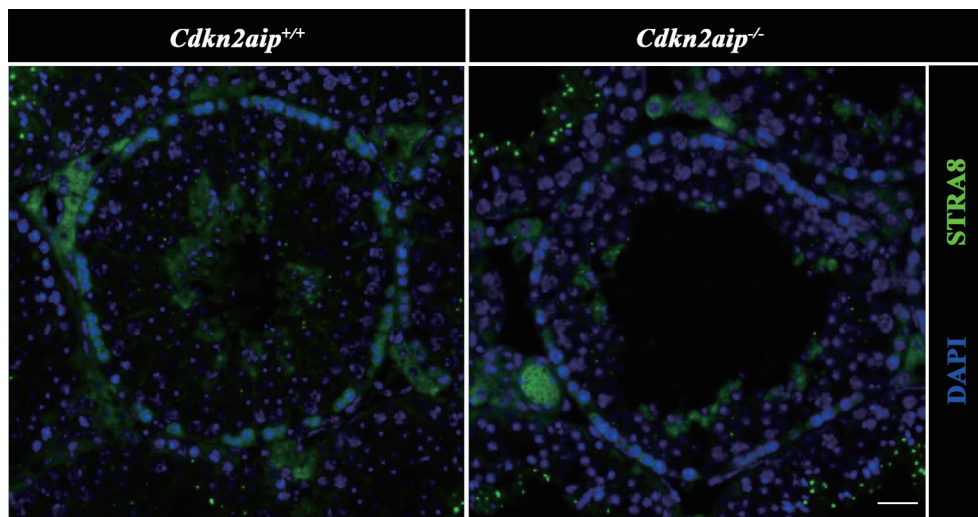

F

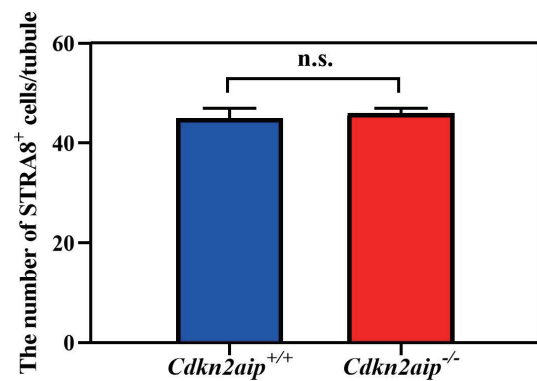

Supplement: Supplementary file 2 — Additional file 2: Figure S2. Cdkn2aip-/- mice exhibit normal spermatogonia and Sertoli cells development. (A) Frozen section staining of SOX9 (green) in testicular tissue from P56 Cdkn2aip +/+ and Cdkn2aip-/- mice. Scale bar, 50μm. (B) Statistical results of (A). Data are presented as average percentage, n=3 mice for each group, and 50 tubules were counted for each mouse. (C) Frozen section staining of PLZF (red) in testicular tissue from P56 Cdkn2aip +/+ and Cdkn2aip-/- mice. Scale bar, 50μm. (D) Statistical results of (C). Data are presented as average percentage, n=3 mice for each group, and 50 tubules were counted for each mouse. (E) Frozen section staining of STRA8 (green) in testicular tissue from P56 Cdkn2aip +/+ and Cdkn2aip-/- mice. Scale bar, 50μm. (F) Statistical results of (E). Data are presented as average percentage, n=3 mice for each group, and 50 tubules were counted for each mouse. [file 13578_2022_861_MOESM2_ESM.pdf]

**A**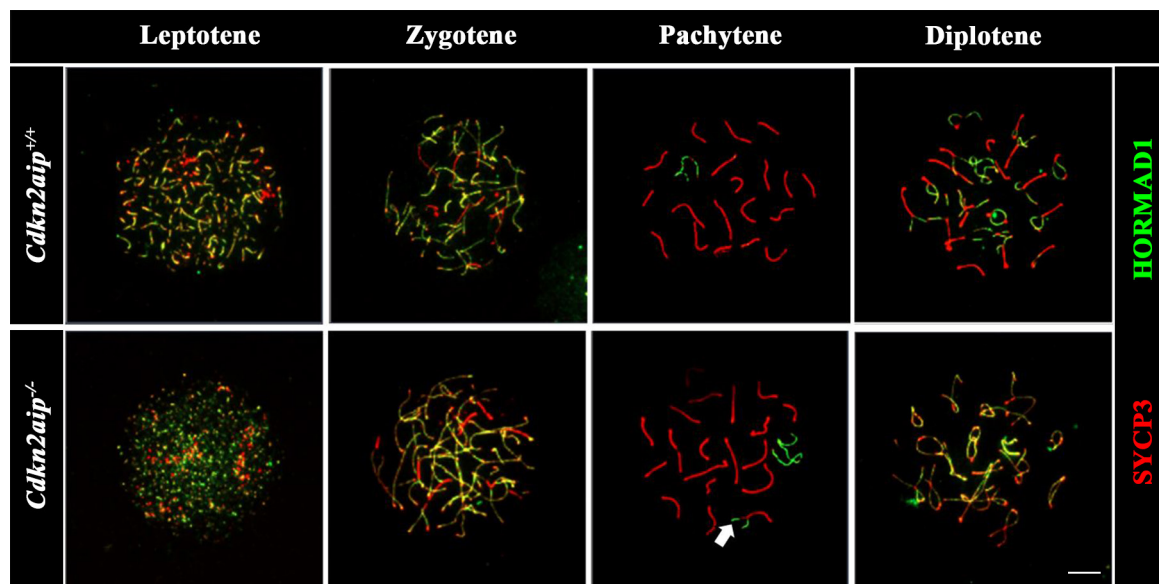**B**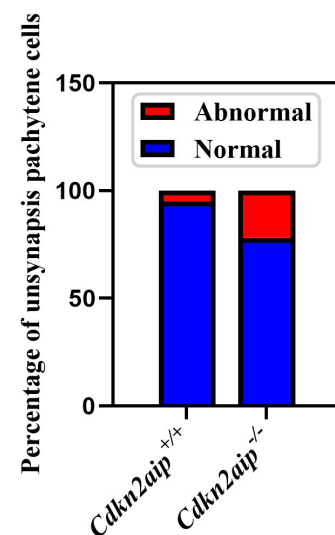**C**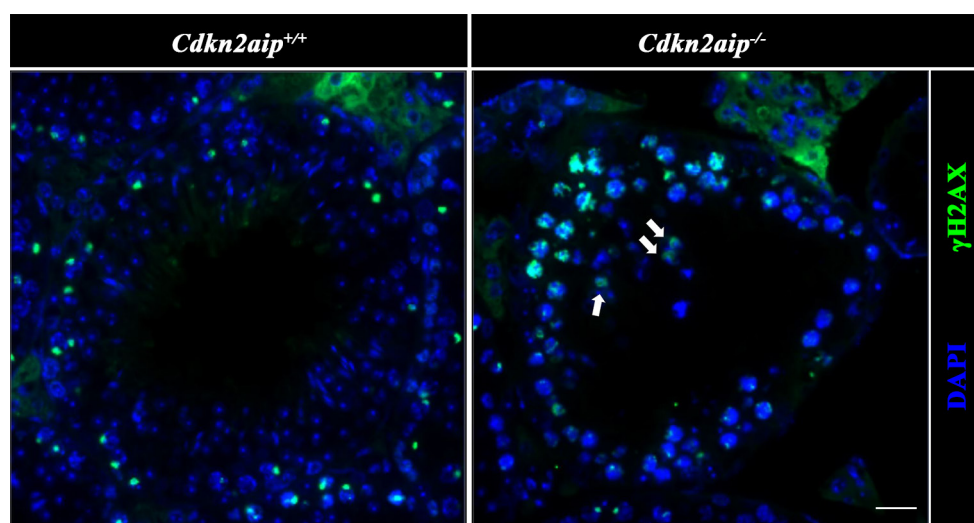**D**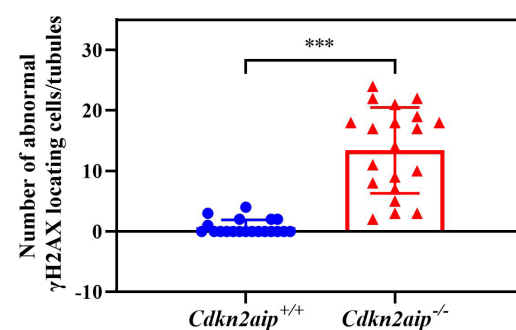**E**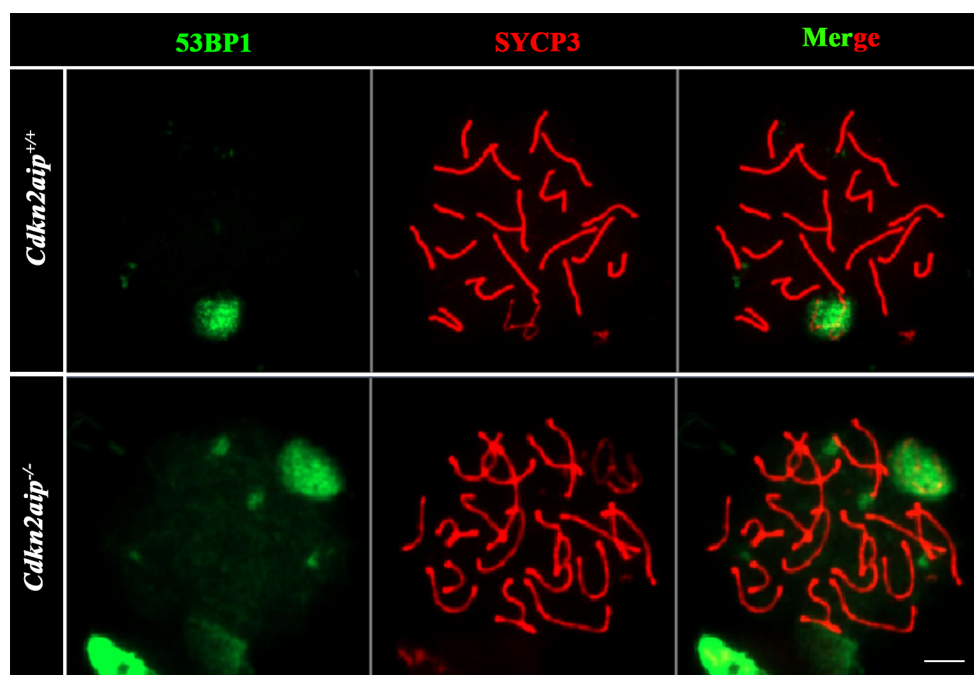**F**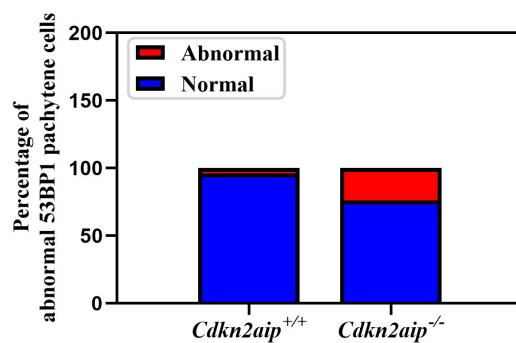

Supplement: Supplementary file 3 — Additional file 3: Figure S3. Cdkn2aip-/- mice exhibit abnormal autosomal synapsis at meiosis prophase I. (A) Immunostaining of SYCP3(red) and HORMAD1(green) in Cdkn2aip +/+ and Cdkn2aip-/- pachytene spermatocytes, n=3 mice for each group Scale bars, 10μm. (B) Statistical results of (A). Data are presented as average percentage, n=3 mice for each group, and 50 spermatocyte were counted for each mouse. (C) Frozen section staining of γH2AX (green) and DIPA (blue) in testicular tissue from P56 Cdkn2aip +/+ and Cdkn2aip-/- mice. Scale bar, 50μm. (D) Statistical results of abnormal γH2AX signals/tubule. n=3 mice for each group, and 20 tubules were counted for each mouse. Data are presented as mean±SD, ***P<0.001 by two-tailed Student’s-test. (E) Immunostaining of 53BP1 (green) and SYCP3(red) and on chromosome spreads of spermatocytes from P35 Cdkn2aip +/+ and Cdkn2aip-/- mice testes, n=3 mice for each group. Scale bars, 10μm. (F) Statistical analysis of abnormal location percentage of 53BP1 in pachytene spermatocytes. Data are presented as average percentage, n=3 mice for each group, and 100 pachytene spermatocytes were counted for each mouse. [file 13578_2022_861_MOESM3_ESM.pdf]

A

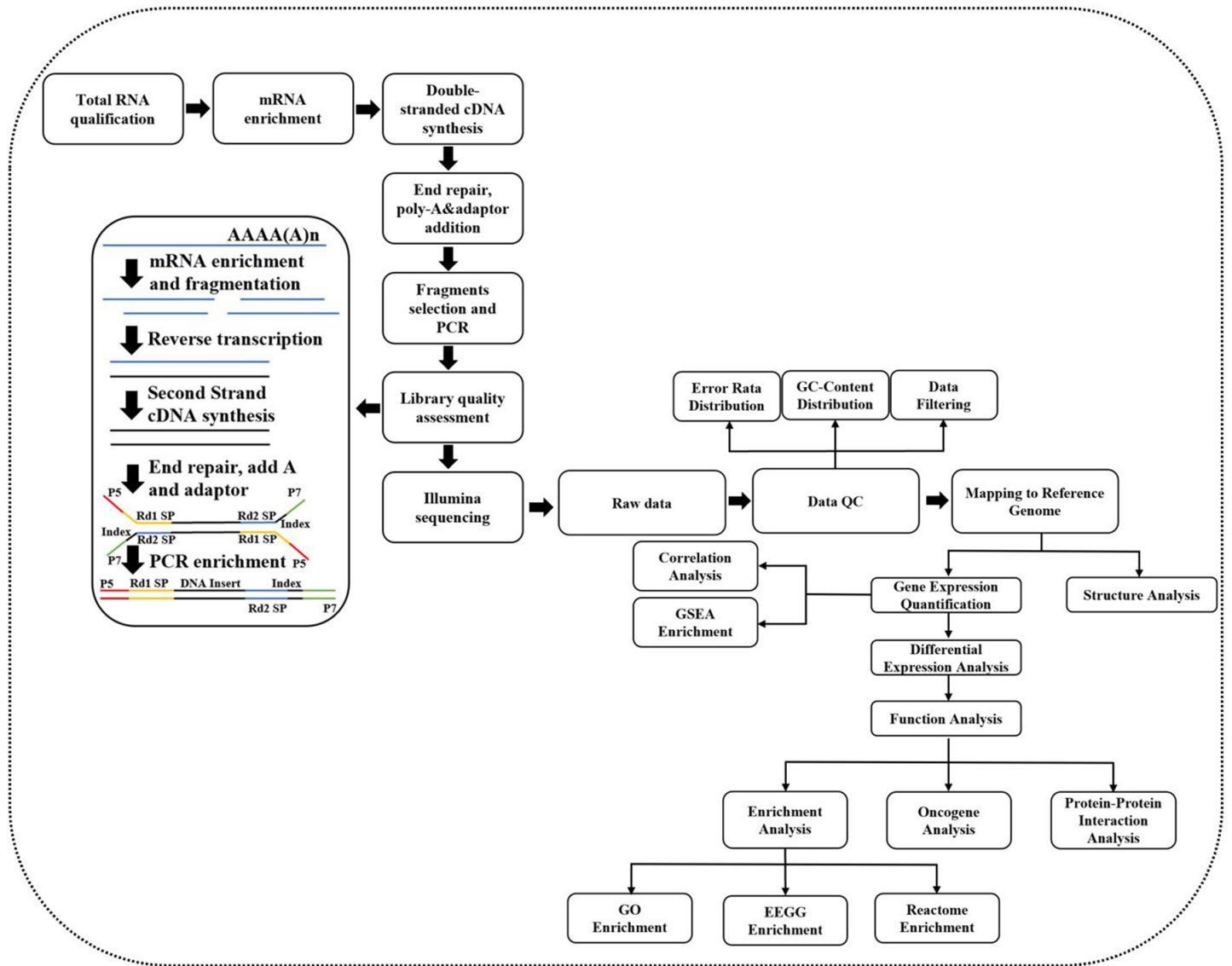

B

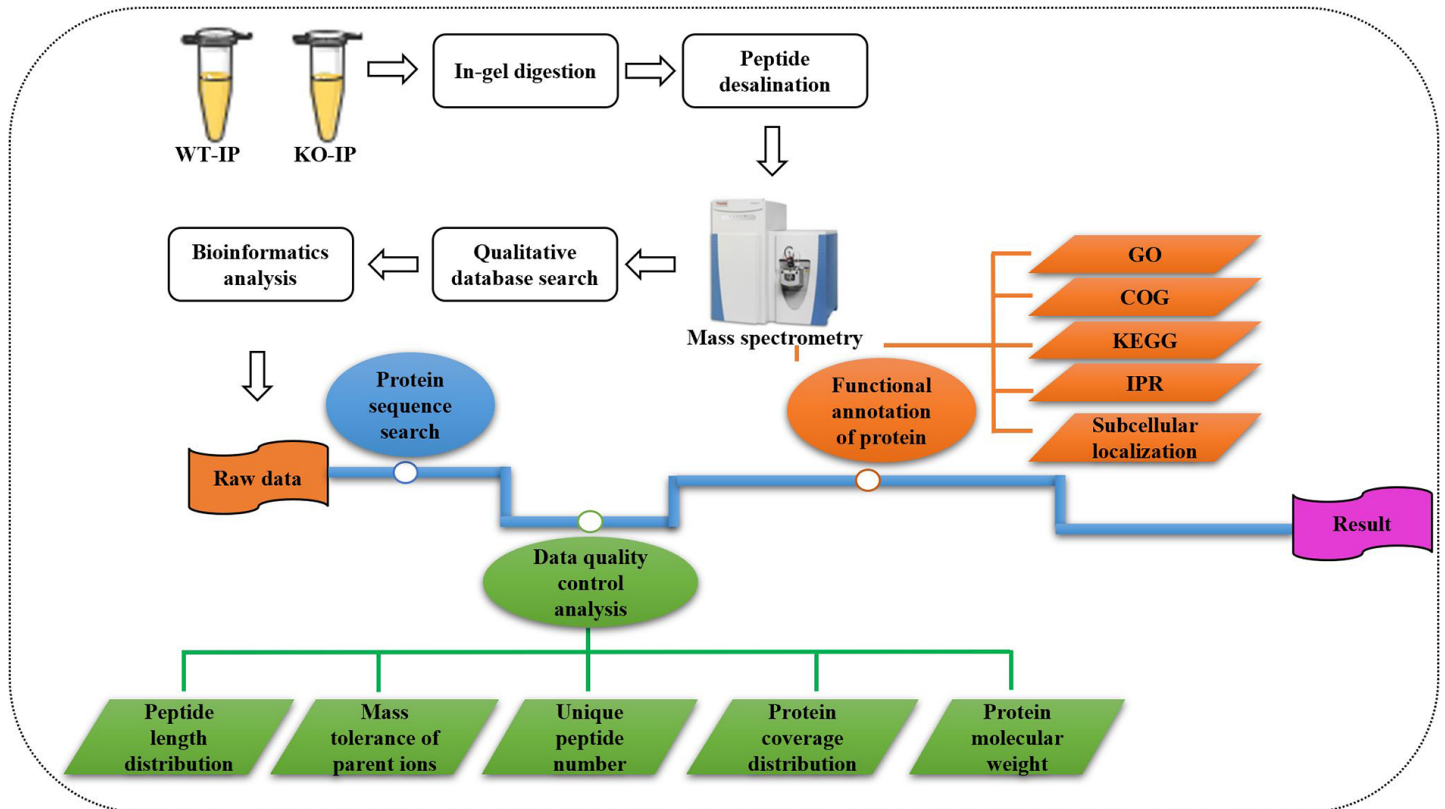

Supplement: Supplementary file 4 — Additional file 4: Figure S4. Principle and data analysis process of RNA Seq and IP-MS. (A) The details of experimental operation and data analysis of RNA seq. (B) The details of experimental operation and data analysis of IP-MS. [file 13578_2022_861_MOESM4_ESM.pdf]

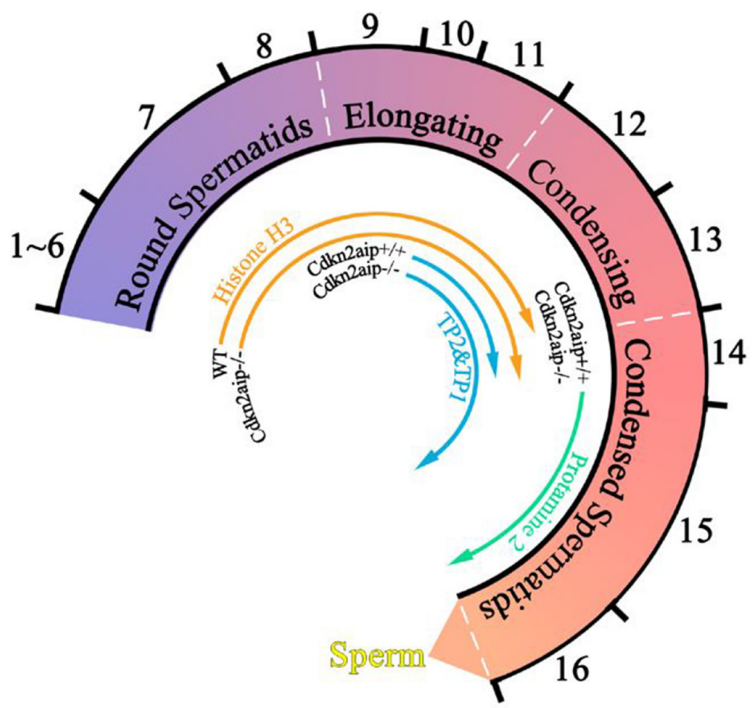

Supplement: Supplementary file 5 — Additional file 5: Figure S5. Schematic diagram showing the proposed model of CDKN2AIP function during spermatogenesis in mice [file 13578_2022_861_MOESM5_ESM.pdf]
