# Supplementary material for: CDKN2AIP is critical for spermiogenesis and germ cell development
Source: Cell Biosci. 2022 Aug 21;12:136. doi: 10.1186/s13578-022-00861-z (PMC9394077; doi:10.1186/s13578-022-00861-z)
Supplement: Supplementary file 6 — Additional file 6: Table S1. Primer sequences are used in this study. [file 13578_2022_861_MOESM6_ESM.docx]

**Figure S1. Targeted knock-out of the *Cdkn2aip* Gene resulted in age-dependent infertility in male mice**

(A) qPCR analyses of *Cdkn2aip* mRNA levels in multiple organs in mice, β-actin is serves as a housekeeping gene.

(B) Litter sizes from wild type females mated with either *Cdkn2aip^+/+^* and *Cdkn2aip^−/−^* mice in 14-week-old (n = 6/group). Data are presented as mean ±S.D. Student’s t test; ***p <0.001.

(C) Testicular sections of 14-week-old *Cdkn2aip^+/+^* and *Cdkn2aip^-/-^* were stained with H&E. Scale bar, 50 μm.

(D) CDKN2AIP full-length protein was purified and verified by SDS electrophoresis. (E) Statistical results of the number of seminiferous tubules with less or without germ cell. n=3 mice for each group, and 100 tubules were counted for each mouse.

(F) Relative number of epididymal sperm number of 14-week-old *Cdkn2aip* *^+/+^* and *Cdkn2aip^-/-^* mice (n=6). Data are presented as mean ±S.D. Student’s t test; ***p <0.001.

**Figure S2. *Cdkn2aip^-/-^* mice exhibit normal spermatogonia and Sertoli cells development**

(A) Frozen section staining of SOX9 (green) in testicular tissue from P56 *Cdkn2aip* *^+/+^* and *Cdkn2aip^-/-^* mice. Scale bar, 50μm.

(B) Statistical results of (A). Data are presented as average percentage, n=3 mice for each group, and 50 tubules were counted for each mouse.

(C) Frozen section staining of PLZF (red) in testicular tissue from P56 *Cdkn2aip* *^+/+^* and *Cdkn2aip^-/-^* mice. Scale bar, 50μm.

(D) Statistical results of (C). Data are presented as average percentage, n=3 mice for each group, and 50 tubules were counted for each mouse.

(E) Frozen section staining of STRA8 (green) in testicular tissue from P56 *Cdkn2aip* *^+/+^* and *Cdkn2aip^-/-^* mice. Scale bar, 50μm.

(F) Statistical results of (E). Data are presented as average percentage, n=3 mice for each group, and 50 tubules were counted for each mouse.

**Figure S3. *Cdkn2aip^-/-^* mice exhibit abnormal autosomal synapsis at meiosis prophase I**

(A) Immunostaining of SYCP3(red) and HORMAD1(green) in *Cdkn2aip* *^+/+^* and *Cdkn2aip^-/-^* pachytene spermatocytes, n=3 mice for each group Scale bars, 10μm.

(B) Statistical results of (A). Data are presented as average percentage, n=3 mice for each group, and 50 spermatocyte were counted for each mouse.

(C) Frozen section staining of γH2AX (green) and DIPA (blue) in testicular tissue from P56 *Cdkn2aip* *^+/+^* and *Cdkn2aip^-/-^* mice. Scale bar, 50μm.

(D) Statistical results of abnormal γH2AX signals/tubule. n=3 mice for each group, and 20 tubules were counted for each mouse. Data are presented as mean±SD, ^***^P<0.001 by two-tailed Student’s-test.

(E) Immunostaining of 53BP1 (green) and SYCP3(red) and on chromosome spreads of spermatocytes from P35 *Cdkn2aip* *^+/+^* and *Cdkn2aip^-/-^* mice testes, n=3 mice for each group. Scale bars, 10μm.

(F) Statistical analysis of abnormal location percentage of 53BP1 in pachytene spermatocytes. Data are presented as average percentage, n=3 mice for each group, and 100 pachytene spermatocytes were counted for each mouse.

**Figure S4. Principle and data analysis process of RNA Seq and IP-MS**

(A) The details of experimental operation and data analysis of RNA seq.

(B) The details of experimental operation and data analysis of IP-MS.

**Figure S5. Schematic diagram showing the proposed model of CDKN2AIP function during spermatogenesis in mice**

| **Table S1. Primer sequences are used in this study** | | |
| --- | --- | --- |
| Target | Sequence (5' to 3') | Application |
| Cdkn2aip | F: CTTTTGTTTATTTTGGCCGCGTG | Genotyping |
|  | R1: TAGAAAGCCAGAAGGCCAAAAGTC |  |
|  | R2: CCCTAGGAAGACATGGTTCGC |  |
| β-actin | F:CTTAGTTGCGTTACACCCTTTC | RT-PCR |
|  | R:CACCTTCACCGTTCCAGTTT |  |
| Sun1 | F: CCTGATTCGTGAGCAGACCA | RT-PCR |
|  | R: GTGTATCCATTGCTGCTCGC |  |
| Prm1 | F: ATGGCCAGATACCGATGCTG | RT-PCR |
|  | R: GCAGCATCTTCGCCTCCTC |  |
| Prm2 | F: GAGCGCGTAGAGGACTATGG | RT-PCR |
|  | R: ATCTTCTGCAGCCTCTGCGAT |  |
